# Redox and Reactive Oxygen Species Network in Acclimation for Salinity Tolerance in Sugar Beet

**DOI:** 10.1093/jxb/erx019

**Published:** 2017-02-18

**Authors:** M Sazzad Hossain, Abdelaleim Ismail ElSayed, Marten Moore, Karl-Josef Dietz

**Affiliations:** 1 Department of Biochemistry and Physiology of Plants, Faculty of Biology, University of Bielefeld, D-33501 Bielefeld, Germany; 2 Biochemistry Department, Faculty of Agriculture, Zagazig University, 44519 Zagazig, Egypt

**Keywords:** Alternative oxidase, antioxidant defense, NADPH oxidase, peroxiredoxin, RBOH, salinity stress, sugar beet, superoxide dismutase

## Abstract

Fine-tuned and coordinated regulation of transport, metabolism and redox homeostasis allows plants to acclimate to osmotic and ionic stress caused by high salinity. Sugar beet is a highly salt tolerant crop plant and is therefore an interesting model to study sodium chloride (NaCl) acclimation in crops. Sugar beet plants were subjected to a final level of 300 mM NaCl for up to 14 d in hydroponics. Plants acclimated to NaCl stress by maintaining its growth rate and adjusting its cellular redox and reactive oxygen species (ROS) network. In order to understand the unusual suppression of ROS accumulation under severe salinity, the regulation of elements of the redox and ROS network was investigated at the transcript level. First, the gene families of superoxide dismutase (SOD), peroxiredoxins (Prx), alternative oxidase (AOX), plastid terminal oxidase (PTOX) and NADPH oxidase (RBOH) were identified in the sugar beet genome. Salinity induced the accumulation of *Cu-Zn-SOD*, *Mn-SOD*, *Fe-SOD3*, all *AOX* isoforms, *2-Cys-PrxB*, *PrxQ*, and *PrxIIF*. In contrast, *Fe-SOD1*, *1-Cys-Prx*, *PrxIIB* and *PrxIIE* levels decreased in response to salinity. Most importantly, *RBOH* transcripts of all isoforms decreased. This pattern offers a straightforward explanation for the low ROS levels under salinity. Promoters of stress responsive antioxidant genes were analyzed *in silico* for the enrichment of *cis*-elements, in order to gain insights into gene regulation. The results indicate that special *cis*-elements in the promoters of the antioxidant genes in sugar beet participate in adjusting the redox and ROS network and are fundamental to high salinity tolerance of sugar beet

## Highlight

Sugar beet acclimation to high salinity stress involves the down-regulation of reactive oxygen species generator systems and the up-regulation of antioxidant enzymes.

## Introduction

Sugar beet (*Beta vulgaris* L.) has become an important source for sugar production in temperate areas of the world. It is not only used in the food industry but also for the production of bioethanol as a source of renewable energy (Magaña *et al.*, 2011). Sugar beet is considered to be a cash crop and requires careful agronomical practices and breeding for adaptation to biotic and abiotic stresses. It is cultivated in different climates in Europe, North America, and increasingly in Asia, South America and recently North Africa. This suggests that bred cultivars are able to cope with different environments and growth conditions. Particular traits of interest for improving sugar beet production include its ability to acclimate to biotic and abiotic stresses, such as cold in temperate climates as well as drought, heat and salinity (Vastarelli *et al.*, 2013).

Drought and salinity are among the most serious threats for crop production and limit agricultural productivity around the world (Horie and Schroeder, 2004; Munns and Tester, 2008). Saline soils are widespread, which can in part be attributed to the common global issue of water deficiency (Turan *et al.*, 2009). Enhanced salinity tolerance will enable more productive use of saline soil and hence mechanisms involved in this ability are important areas of plant research (Horie and Schroeder, 2004; Hussain *et al.*, 2008; Katori *et al.*, 2010). High salt concentrations in rooting medium induce ionic and osmotic imbalances and oxidative damage, which results in growth retardation, wilting or death (Parida *et al.*, 2004). Successful acclimation includes physiological and biochemical changes (Taji *et al.*, 2004). Selective ion uptake, exclusion and compartmentalization are required to maintain a proper K^+^/Na^+^-balance while synthesis of compatible solutes, such as glycine, betaine and proline, is also needed (Yeo, 1998; Hamouda *et al.*, 2016). Analysis of the signaling pathways and regulatory mechanisms involved indicates that hormones, Ca^2+^, and redox cues function as central players in acclimation (Zhang *et al.*, 2008). Sugar beet tolerates salinity of up to 500 mM sodium chloride (NaCl) for 7 d without losing viability (Yang *et al.*, 2012). The genome sequence of sugar beet was recently reported (Dohm *et al.* (2014), making sugar beet an excellent model for studying plant response and tolerance to salinity stress (Yang *et al.*, 2012).

Salinity stress affects cellular reactive oxygen species (ROS) generator systems, such as photosynthetic electron transport, photorespiratory hydrogen peroxide (H_2_O_2_) release, respiratory electron transport, and enzyme activities including glucose oxidase, xanthine oxidase and in particular, plant peroxidases and NADPH oxidase. One of the most important cellular ROS generating systems is the plasma membrane-bound NADPH oxidase RBOH (Keller *et al.*, 1998), which decisively controls cellular redox homeostasis under salinity stress (Hossain and Dietz, 2016). ROS accumulation is a common denominator under conditions of stress (Foyer *et al.*, 1994).

Complementary to the generator systems are enzymatic and non-enzymatic antioxidants that regulate ROS and redox homeostasis and counteract metabolic imbalances, damage to cell structures, cell death and stress adaptation (Foyer *et al.*, 2009). Superoxide dismutases (SODs) constitute the first line of defense against superoxide (O_2_•^−^), which is produced in most cellular compartments (Elstner, 1991). SODs are therefore found in all subcellular compartments (Alscher *et al.*, 2002). H_2_O_2_ is decomposed by thiol peroxidases, in particular glutathione peroxidases (GPXs) and peroxiredoxins (Prxs), and by type III heme peroxidases, ascorbate peroxidase (APX) and catalase (Mittler and Poulos, 2005). GPXs and Prxs decompose alkyl hydroperoxides in addition to H_2_O_2_ and employ a thiol mechanism to react with peroxide (Dietz, 2016). Following oxidation by the peroxide substrate, thiol peroxidases are regenerated by electron donors such as thioredoxins, glutaredoxins and glutathione (Navrot *et al.*, 2006; Koh *et al.*, 2007; Dietz, 2011). Prxs display a stable TRX-fold and are grouped into four subfamilies: 1-CysPrx, 2-CysPrx, PrxQ and PrxII-like proteins (Horling *et al.*, 2003; Muthuramalingam *et al.*, 2009). Prxs and GPXs play important roles as thiol antioxidants, modulators of cell signalling and redox sensors (Dietz *et al.*, 2006; Navrot *et al.*, 2006).

Here, we identified some of the central components of the cellular antioxidant defense system in sugar beet, and followed their transcriptional response during acclimation to 300 mmol L^-1^ NaCl. We tested the hypothesis that the ROS and redox network participates in the extraordinarily high salinity tolerance of sugar beet. The transcriptional response pattern was placed into the broad framework of the cellular redox state. This study addressed an additional layer of redox and ROS homeostasis, namely the activation of safety mechanisms, as there are terminal oxidases present in different subcellular compartments. Mitochondrial alternative oxidase (AOX) (Considine *et al.*, 2002; Hossain and Dietz, 2016) and plastid terminal oxidase (PTOX) (Stepien and Johnson, 2009; Hossain and Dietz, 2016) help dissipate excess reducing power.

## Materials and methods

### Plant materials and NaCl treatment

**Fig. 1.**
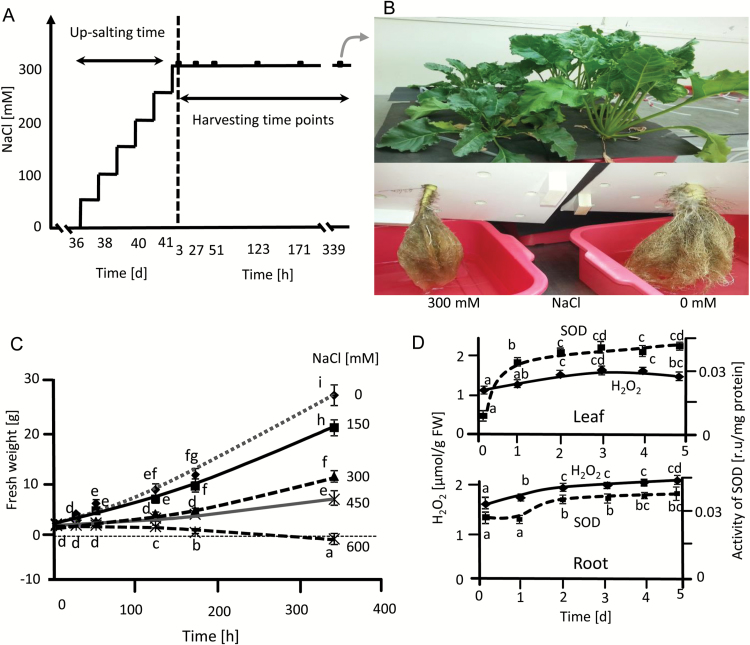
Selection of salinity conditions for sugar beet acclimation experiments. (A) The experimental design consisted of stepwise up-salting to 300 mM NaCl and harvesting after 3 h, or 339 h i.e. a 14-day period. For some analyses four additional time points were used as indicated. (B) Morphology of plants grown in hydroponics with or without 300 mM NaCl for 14 ds, with a total growth period of 55 d. (C) Growth as indicated by fresh weight increase of sugar beet at different NaCl concentrations. Data are means ±SD of *n*=5 experiments with three measurements each. (D) Monitoring of early responses of sugar beet during up-salting, namely H_2_O_2_ levels and SOD activity under control and salinity stress conditions. Data are means ±SD of *n*=5 experiments with six measurements each for H_2_O_2_ and three measurements each for SOD activity. The significant difference marked by different letters was calculated using Student’s *t*-test and further analyzed with Fisher LSD test, with *P*<0.05, using InfoStat statistical software.

Seeds of sugar beet (*Beta vulgaris* subsp. vulgaris), cultivar KWS2320 were sterilized with 70% (v/v) ethanol, 0.1% (w/v) HgCl_2_ and 0.2% (w/v) thiram, placed in germination pots in vermiculite and perlite mixture and soaked in water in darkness for one week. The tray was then placed in growth chambers with 10 h light with an intensity of 100 µmol m^−2^ s^−1^ at 21 °C and 14 h darkness at 18 °C with 55% relative humidity for another week. The growth condition was adjusted according to the temperate climate, as the cultivar is adapted to temperate regions. After 14 d, uniformly grown seedlings were transferred to hydroponic containers with Hoagland solution (Ghoulam *et al.*, 2002). All plants grew under control conditions for 35 d and up-salting started from day 36 in the case of salinity-stressed plants. Salt concentration was gradually increased to 300 mM NaCl in 50 mM^.^d^−1^ increments (Fig. 1A). Tissue was harvested from the fully expanded third to sixth leaves and the whole root after the point of first branching, from five independent experiments at time points indicated. Tissue was then frozen in liquid nitrogen and stored at −80 °C.

### Determination of photosystem II quantum yield and CO_2_ fixation rate

The steady-state quantum yield (F_V_/F_M_) of photosystem II (PSII) was measured using the Mini-PAM Fluorometer (Walz, Germany) under light conditions as described by Oelze *et al.* (2012). CO_2_ fixation of sugar beet leaves under stressful and control conditions was measured with a portable gas exchange system (GFS-3000, Heinz Walz GmbH, Effeltrich, Germany). The CO_2_ assimilation rate was measured at a light intensity of 100 µmol photons m^−2^ s^−1^, a relative humidity of 50% and at 22 °C.

### Determination of osmotic potential and sodium, potassium and chloride content

Osmotic potential was measured by using an automatic cryoscopic osmometer (Knauer, Berlin, Germany), following calibration between 0 and 300 mosmol kg^-1^. The osmotic potential is given as mosmol kg^-1^. Sodium and potassium contents were determined from tissue sap using a flame photometer (Model 410; Sherwood Scientific Ltd, Cambridge, UK) calibrated between 0 and 10 ppm. Chloride content was determined in tissue sap using a Chloride Analyzer (Model 926S; Sherwood Scientific Ltd, Cambridge, UK).

### Determination of antioxidant and non-protein thiols content

Ascorbate (AA) and dehydroascorbate (DHA) content were determined as described by Horling *et al.* (2003). Glutathione (GSH) and oxidized glutathione (GSSG) content were quantified with an enzyme cycling assay based on sequential oxidation of GSH by 5,5’-dithiobis(2-nitrobenzoate) (DTNB) and reduction by NADPH in the presence of glutathione reductase (GR) (Griffith, 1980). Tissue weighing 200 mg was extracted in 1 ml 0.1 M HCl and 0.1 mM EDTA. For total GSH content, 200 µL neutralized supernatant was incubated with 6 mM DTNB for 5 min followed by a 15 min incubation with 5 µL 2-vinylpyridine. After centrifugation, total GSH content was determined from the supernatant. The reaction was started with the addition of GR. Changes in 5’-thio-2-nitrobenzoic acid absorbance were spectrophotometrically monitored at 412 nm. To determine GSSG content, the neutralized supernatant was incubated first with 2-vinylpyridine for 15 min, then with 6 mM DTNB for 5 min and subsequently GR and NADPH were added. The difference between total glutathione and GSSG content is presented as GSH content. Non-protein thiols content was determined using 0.1 M phosphate buffer at pH 7.0, 0.5 mM EDTA and 1 mM DTNB (Del Longo *et al.*, 1993). Absorbance was measured at 412 nm using a microplate reader (KC4, BIOTEK Instruments, Bad Friedrichshall, Germany). Values were corrected for the absorbance of a blank without extract and determined with a glutathione standard curve.

### Determination of protein, malondialdehyde and hydrogen peroxide content

Total protein (mg g^-1^ FW) content was determined using bovine serum albumin as a standard, according to Bradford (1976). H_2_O_2_ content was quantified by chemiluminescence with luminol (Pèrez and Rubio, 2006). Malondialdehyde (MDA) content was measured colorimetrically according to Stewart and Bewley (1980) with some modification. Leaf tissue weighing 100 mg was homogenized in 0.1% trichloroacetic acid (TCA) on ice. Following centrifugation, 1.5 ml of 20% TCA containing 0.5% thiobarbituric acid (TBA) was added to 500 ml supernatant and incubated at 95 °C for 30 min. Following cooling and centrifugation, absorption was measured at 532 nm and the amount of MDA calculated, with ε=155 mM^−1^ cm^−1^.

### SDS-PAGE and immunoblotting

SDS-PAGE and western blotting were performed as in Ströher *et al.* (2009). Binding of the first rabbit antibodies was achieved overnight in 1% skimmed milk in Tris-buffered saline with 0.1% Tween 20 (TBST) at the following dilutions: anti-At2-CysPrx, PrxQ and CuZn SOD2 at 1:3000; anti-*Avena sativa* D1 at 1: 5000. After secondary antibody binding, proteins were detected using chemiluminescence.

### Antioxidant enzyme activities

Tissue weighing 200 mg was homogenized in 1 ml of 0.1 M phosphate buffer at pH 6.5. The supernatant was used to determine the activity of enzymes according to Urbanek *et al.* (1991) and protein content as above (Bradford, 1976). The reaction mixture for catalase consisted of 100 µl extract in 3 ml phosphate buffer at pH 6.8 (Urbanek *et al.*, 1991). The detoxification of H_2_O_2_ was measured at 240 nm (Cary 300 Bio UV/VIS, Varian, Middelburg, Netherland), with ε=43.6 M cm^−1^. SOD activity was quantified according to Sen Gupta *et al.* (1993) and peroxidase according to Malik and Singh (1980), with ε=25 mM^−1^ cm^−1^. APX activity was assessed according to Yoshimura *et al.* (2000) by monitoring the rate of ascorbate oxidation at 290 nm, with ε=2.8 mM^−1^ cm^−1^. GR activity was measured by following the increase in absorbance in the presence of GSSG and DTNB (Sairam *et al.*, 2002), with ε=13.6 mM^−1^ cm^−1^.

### Determination of plasma membrane NADPH oxidase activity

NADPH-dependent O_2_^−^ generating activity in isolated microsomal membrane vesicles was determined by following the O_2_^−^-dependent reduction of XTT (Na 3′-[1-[phenylaminocarbonyl]-3,4-tetrazolium]-bis(4-methoxy-6-nitro)benzenesulfonic acid) (Kaundal *et al.*, 2012). The assay mixture contained 50 mM Tris-HCl at pH 7.5, 0.5 mM XTT, 0.1 mM NADPH and the membrane fraction. Tissue was ground in liquid nitrogen and 0.5 g of the powder was weighed out in empty pre-chilled Falcon tubes. 6 ml of ice-cold protein extraction buffer was added on ice and vortexed at room temperature. Homogenized tissue was filtered through four layers of cheesecloth and the flow through transferred to 2 ml microcentrifuge tubes on ice. After centrifugation at 10 000*g* at 4 °C for 45 min, the supernatant was transferred to ultracentrifuge tubes. Microsomal membranes were pelleted from the supernatant by centrifugation at 50 000*g* for 30 min. The pellet was suspended in 0.33 M sucrose, 3 mM KCl, and 5 mM potassium phosphate at pH 7.8. The plasma membrane fraction was isolated by adding the microsomal suspension to an aqueous two-phase polymer system to give a final composition of 6.2% (w/w) Dextran T500, 6.2% (w/w) polyethylene glycol (PEG) 3000, 0.33 M sucrose, 3 mM KCl, and 5 mM potassium phosphate at pH 7.8 with protease inhibitors. After three rounds of partitioning the resulting upper phase was diluted 5-fold in ice cold 10mM Tris-HCl dilution buffer at pH 7.4, containing 0.25 M sucrose, 3 mM EDTA, 1 mM DTT, 3.6 mM L-cysteine, 0.1 mM MgCl_2_ and protease inhibitors. The fractions were centrifuged at 120000*g* for 30 min. The pellets were resuspended in 1 ml of 10 mM Tris-HCl at pH 7.4 for the activity assay. All procedures were carried out at 4 °C. The reaction was initiated with NADPH. In the presence of O_2_^−^, XTT generates a yellow formazan that was quantified spectrophotometrically at 492 nm and calculated with ε=21 600 M^−1^ cm^−1^.

### Transcript quantification

RNA isolation and cDNA synthesis were performed according to Wormuth *et al.* (2006) with a few modifications. Semi-quantitative RT-PCR analysis was carried out as previously described at the individually optimized cycle number (Finkemeier *et al.*, 2005), using the given primer combinations (see Supplementary Table S1 at *JXB* online). Primers were designed with Primer3Plus software (www.bioinformatics.nl/cgi-bin/primer3plus). Loading was normalized with actin. qRT-PCR was carried out in the iCycler ™ Thermal Cycler (Bio-Rad, USA) with the iQ™SYBR Green Supermix (Bio-Rad, USA) in a final volume of 20 µl according to the manufacturer’s instructions. The iCycler was programmed to 95 °C for 1 min; 45x (95 °C for 30s, 58 °C for 40s, 72 °C for 45 s), 72 °C for 10 min, followed by a melting curve program of 55–95 °C in increasing steps of 0.5 °C. Efficiencies of each reaction were calculated using LinRegPCR software (Ruijter *et al.*, 2009). Signal values were subsequently derived from the threshold cycles, with the average background subtracted, using the equation provided by Pfaffl (2001).

### Sequence alignment and construction of phylogenetic trees

Sugar beet genes homologous to known *Arabidopsis thaliana* SOD, Prx, AOX and RBOH genes were searched for using FASTA and WU-BLAST2 programs. The amino acid sequences were aligned using CLUSTALW 2.1 (Larkin *et al.*, 2007) with the default configuration. Their phylogenetic relationships were determined using the maximum likelihood (ML) algorithm incorporated in the program MEGA version 5 (Tamura *et al.*, 2011). Bootstrap analyses with 500 replicates were performed to assess the robustness of the branches. Pairwise sequence alignment was done by blasting the isoforms of each gene group and sequence identity was retrieved (see Supplementary Table S2).

### *Cis*-regulatory elements analysis

Promoters of stress-responsive genes in sugar beet were analyzed *in silico* for putative *cis*-elements using available genome sequences and our transcriptomic data. The Plant Promoter Analysis Navigator (PlantPAN 2.0; http://PlantPAN2.itps.ncku.edu.tw) (Chow *et al.*, 2015) and The AthaMap (AthaMap 8; http://www.athamap.de) (Steffens *et al.*, 2005) databases were used to identify *cis*-regulatory elements for 1000 bp upstream promoters of differentially regulated genes of *B. vulgaris* under salinity stress, with *A. thaliana* as reference (Table 3, Supplementary Tables S3–S5).

**Table 3.** *Cis*-elements potentially responsible for the regulation of salinity induced transcripts in *B. vulgaris* but absent in the same gene from *A. thaliana*.

Gene name	*Cis*-regulating elements	*Cis*-regulating elements sequence	Expression matrix
Cu-ZnSOD1; Cu-ZnSOD2; Cu-ZnSOD3; FeSOD3; MnSOD1; 2CysPrx B; Prx Q; AOX1A; AOX2; PTOX1;RBOH B	Dehydrin (LTRECOREATCOR15)	CCGAC	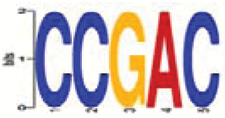
Cu-ZnSOD1; 2CysPrx B; AOX1A; AOX2; PTOX1	BES1 (TFmatrixID_0494)	CACGTG*	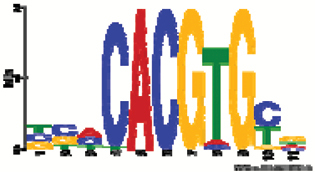

* indicates details provided in Supplementary Table S5.

### Statistical analysis

Pairwise comparisons were performed with Students *t*-test. Statistical grouping of the data was achieved by applying Fisher’s LSD, with *P*<0.05, using InfoStat statistical software.

## Results

### Effect of salinity on growth and redox state of sugar beet

The NaCl concentration in the hydroponics solution was increased stepwise to 300 mM. The plants were then kept at this level for 14 d for detailed kinetic analysis (Fig. 1A). This regime was chosen after comparing different NaCl concentrations, where growth was still measureable at 450 mM but ceased at 600 mM. Morphological changes were observed after salinity treatment (Fig. 1B–C). The high final salt level of 300 mM NaCl still enabled significant growth. The plant response was scrutinized by following the level of H_2_O_2_ and SOD activity during up-salting (Fig. 1D). H_2_O_2_ levels increased in roots and leaves after the first salt step and until the second step but did not increase further during subsequent up-salting steps. Instead levels started to decline at the highest salt concentrations in leaves. Total SOD activity roughly followed H_2_O_2_ levels indicating that sugar beet acclimated to salinity with no effects on fresh weight-related protein content, which was considered important for subsequent analyses (see Supplementary Fig. S1).

### Photosynthetic quantum yield, CO_2_ fixation, ionic and osmotic state

**Fig. 2.**
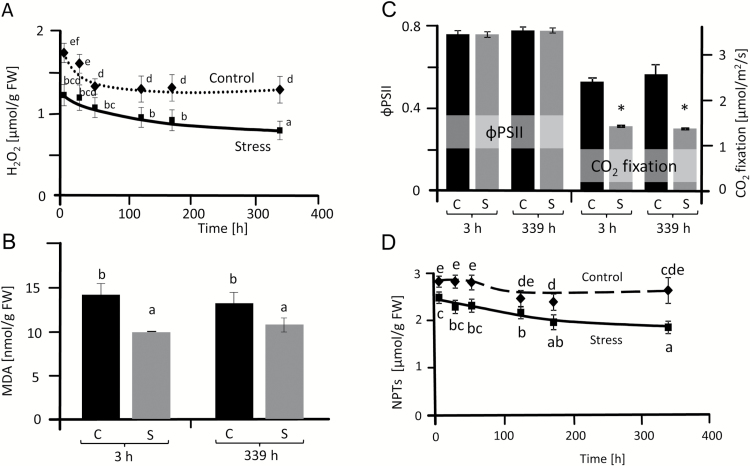
Characterization of ROS and antioxidant status of salt-stressed sugar beet. H_2_O_2_ content (A)_,_ malondialdehyde (MDA) content (B), photosynthetic quantum yield (ΦPSII) and CO_2_ fixation rates (**C**) and non-protein thiols content (D) in controls and plants stressed with 300 mM NaCl. The first analysis was carried out 3 h after reaching a concentration of 300 mM NaCl. Data are means ±SD of *n*=5 experiments with six measurements each for H_2_O_2_ and three for MDA and non-protein thiols. The significant difference marked by different letters was calculated using Student’s *t*-test and further analyzed with Fisher LSD test, with *P*<0.05, using InfoStat statistical software. Along the x-axis: C, control; S, salinity.

The quantum yield of photosystem II (ΦPSII) was unchanged between salt-treated and control plants during up-salting and at different time points at 300 mM NaCl (Fig. 2C, Supplementary Fig. S2A, B). CO_2_ fixation rates decreased under salt treatment with little difference between 3 h and 14 d at 300 mM NaCl (Fig. 2C). This is despite the strong increase in osmotic potential, and sodium, chloride and potassium content (see Supplementary Fig. S2C–F).

### Oxidative stress and antioxidant defense

H_2_O_2_ accumulation, as a recognized marker of oxidative stress, was quantified in leaves during the 14-day period, after reaching 300 mM NaCl (Fig. 2A). The basal steady state level of H_2_O_2_ was around 1.7 and 1.2 nmol mg^-1^ FW in unsalted control and up-salted sugar beet, respectively. The H_2_O_2_ content in sugar beet leaves decreased during aging but importantly it was always significantly lower in salt-treated tissue compared with controls (Fig. 2A). MDA levels in salt-stressed leaves were also below control samples indicating less lipid peroxidation and membrane damage (Fig. 2B). Non-protein thiols (NPTs) were lower in stressed plants than in controls (Fig. 2D). Ascorbate and glutathione pools were analyzed as these low molecular mass antioxidants are linked to redox homeostasis and signaling and also function as reductants in the water–water cycle (Oelze *et al.*, 2012). Glutathione levels dropped in stressed plants from 3 h to 14 d after up-salting (Table 2). Significantly lower GSH levels were observed in stressed plants at 14 d. In contrast there was no significant difference in GSSG levels for control and salinized plants. Significantly lower ascorbate levels were observed in stressed plants at 27, 123, 171 and 339 h. DHA content during stress acclimation was unchanged (Table 2). The reduction state of both metabolites was decreased at final harvest.

**Table 2.** Characterization of glutathione and ascorbate status of control and salt-stressed sugar beet plants The first analysis was done 3 h after reaching a concentration of 300 mM NaCl. Data are means ±SD of *n*=5 experiments with two technical replicates for glutathione and ascorbate. The significant difference was calculated using Student’s *t*-test and labelled with different letters after analysis with Fisher LSD test, with *P*<0.05, using InfoStat statistical software.

Time (h)	GSH (µmol g^-1^ FW)		GSSG (µmol g^-1^ FW)		ASC (µmol g^-1^ FW)		DHA (µmol g^-1^ FW)	
	Control	Stress	Control	Stress	Control	Stress	Control	Stress
3	1.83 ± 0.27 a	1.77 ± 0.31 a	0.39 ± 0.12	0.50 ± 0.12	1.65 ± 0.16 d	1.57 ± 0.18 d	0.34 ± 0.19	0.32 ± 0.09
27	1.70 ± 0.28 a	1.69 ± 0.31 a	0.46 ± 0.11	0.39 ± 0.08	1.74 ± 0.19 d,e	1.42 ± 0.07 b,c	0.25 ± 0.12	0.28 ± 0.09
51	1.59 ± 0.38 a	1.57 ± 0.34 a	0.38 ± 0.12	0.44 ± 0.11	1.76 ± 0.15 d,e	1.52 ± 0.13 c,d	0.21 ± 0.11	0.22 ± 0.10
123	1.60 ± 0.26 a	1.41 ± 0.27 a	0.34 ± 0.15	0.32 ± 0.09	1.73 ± 0.16 d,e	1.32 ± 0.15 b	0.25 ± 0.17	0.26 ± 0.11
171	1.63 ± 0.29 a	1.33 ± 0.21 a	0.31 ± 0.18	0.37 ± 0.11	1.57 ± 0.08 d	1.34 ± 0.06 b	0.18 ± 0.11	0.23 ± 0.10
339	1.65 ± 0.16 a	1.21 ± 0.11 b	0.29 ± 0.12	0.25 ± 0.09	1.54 ± 0.15 d	1.25 ± 0.05 a	0.21 ± 0.11	0.16 ± 0.08

### Regulation of peroxiredoxin and superoxide dismutase proteins

**Fig. 3.**
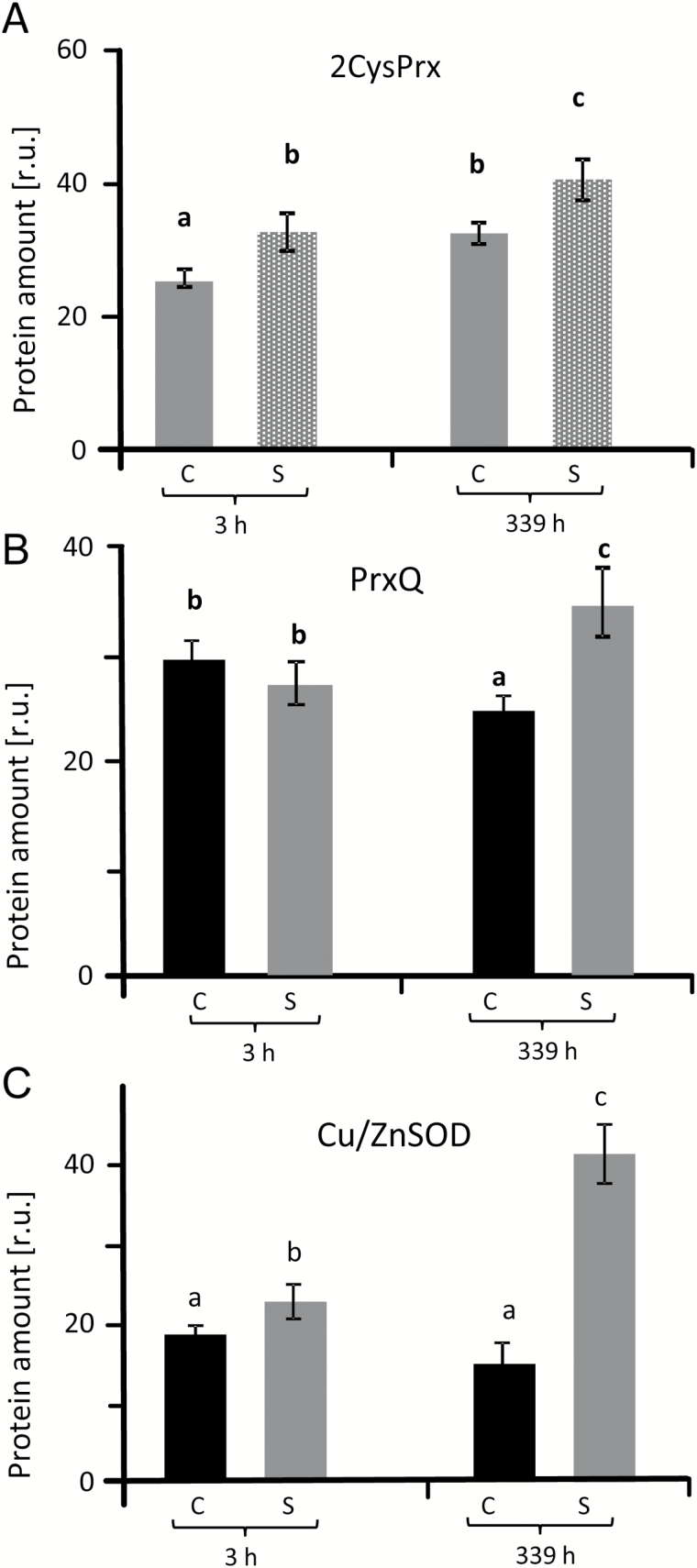
Protein amounts of 2-CysPrx (A), PrxQ (B) and CuZnSOD (C) under salinity stress and in control sugar beet. Band intensities in western blots were densitometrically quantified in three blots from independent experiments (as shown in Supplementary Fig. S3) using ImageJ software (http://imagej.nih.gov/ij/). The significant difference was calculated using Student’s *t*-test and labelled with different letters after analysis with Fisher LSD test, with *P*<0.05, using InfoStat statistical software. C, control; S, salinity.

The abundance of 2-cysteine peroxiredoxin (2-CysPrx) protein was examined via western blotting using control and salt-stressed plant samples (Fig. 3A, Supplementary Fig. S3). 2-CysPrx amounts increased with aging and under salt stress. Chloroplast peroxiredoxin Q (PrxQ) levels were unchanged in salt-treated plants compared with controls at 3 h but were increased after 14 d (Fig. 3B). CuZnSOD showed the strongest response to salt stress with a 3-fold increase after 14 d (Fig. 3C, Supplementary Fig. S3A–C).

### Sugar beet *SOD* gene family members and their transcriptional regulation

**Fig. 4.**
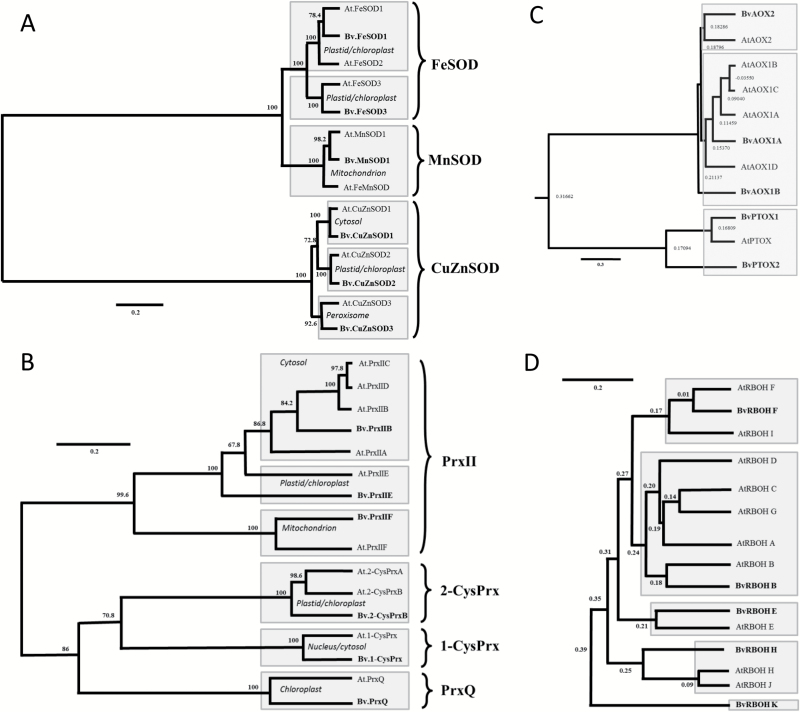
Phylogenetic relationships among SOD, Prx, AOX, PTOX, and RBOH proteins in Arabidopsis and sugar beet. (A) Dendrogram depicting amino acid sequence-based phylogenetic relationships among 14 different SOD isoforms from *B. vulgaris* KWS2320 and *A. thaliana* available in databases. The scale bar shows the number of substitutions per amino acid. Bootstrap analysis of 500 replicates was performed. (B–D) Dendrograms depicting phylogenetic relationships among Prx isoforms (B), AOX/PTOX (C), and RBOHs (D) in sugar beet and Arabidopsis. The numbers above the branches indicate the bootstrap confidence value. The scale bar shows the number of substitutions per amino acid. Bootstrap analysis of 500 replicates was performed. Sugar beet nomenclature is derived from the Arabidopsis nomenclature, considering the closest homologs. If there was a group of related At-sequences, then the letter adopted was that which came first in the alphabet. Two AOX/PTOX and RBOH sequences, each with significant similarity to Arabidopsis genes, received numbers and letters, respectively, which were not yet used in the Arabidopsis system. For gene names and accession numbers please consult Table 1. Sequence identities between different isoforms of the same gene group of sugar beet are provided in Supplementary Table S2.

**Fig. 5.**
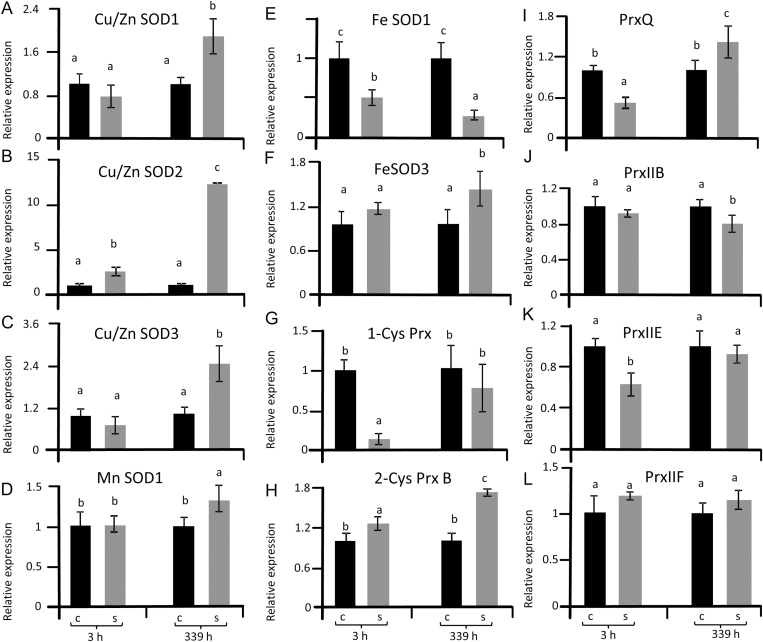
Transcript levels of SOD and Prx in sugar beet under salt stress compared to controls. Transcript amounts were quantified by qPCR at 3 h and 339 h after reaching a concentration of 300 mM NaCl and were calculated relative to actin levels. qPCR data represent the average of three independent experiments with two technical replicates each. The significant difference was calculated using Student’s *t*-test and labelled with different letters after analysis with Fisher LSD test, with *P*<0.05, using InfoStat statistical software. C, control; S, salinity.

The accumulation of 2-Cys-Prx, PrxQ and CuZnSOD proteins under salinity prompted us to use bioinformatics to identify important antioxidant genes in the sugar beet genome and to quantify their transcriptional response by qPCR. Three *CuZnSOD* genes, one *MnSOD* gene and two *FeSOD* genes were identified (Table 1). The construction of a phylogenetic tree showed that the SOD family, with 14 available accessions in the databanks of *B. vulgaris* and *A. thaliana*, segregated into three clearly separated groups based on sequence similarity (Fig. 4A). Group I encompassed *FeSOD* genes that included two isoforms in sugar beet. *MnSOD* isoforms were related to *FeSOD* genes but separated into a distinct cluster. The three *CuZnSOD* genes in sugar beet were grouped in pairs with their cognate homologs in Arabidopsis. Relatedness was confirmed by sequence alignment (Supplementary Fig. S4). With the exception of *FeSOD1*, which was strongly down-regulated under salinity, all other SOD transcripts revealed significantly higher levels at 14 d of salt stress (Fig. 5A–F). Up-regulation was greatest for *SOD2* with approximately a 13-fold difference between 300 mM and 0 mM NaCl (Fig. 5B). Mitochondrial *MnSOD1* transcript levels were unchanged at 3 h but significantly increased at 14 d (Fig. 5D). The order of transcript up-regulation was: *CuZnSOD2* > *CuZnSOD3* > *CuZnSOD1* > *FeSOD3* > *MnSOD.*

**Table 1.** Gene annotation of SODs, Prxs, AOXs and RBOHs in *B. vulgaris* subsp. *vulgaris* genotype KWS2320 and *Arabidopsis thaliana* The table depicts the protein names and genome annotations, putative localization, protein length and calculated molecular mass. Abbreviations: cp, chloroplast; ecm, extracellular matrix, apoplast; mt, mitochondrion; per, peroxisome; vac, vacuole; cyt, cytoplasm; nuc, nucleus; pl, plastid; plast, plastoglobule; pm, plasma membrane; cw, cell wall.

Enzyme and reaction	Protein/gene name		Reference sequence		Localization	Length (amino acids) and mol. wt (kDa)	
	*B*. *vulgaris*	*A*. *thaliana*	*B*. *vulgaris*	*A*. *thaliana*		*B. vulgaris*	*A. thaliana*
Superoxide dismutase (SOD) O_2_^−^+O_2_^−^+2H^+^→H_2_O_2_+O_2_	FeSOD1	FeSOD1	XM_010678412.1	AT4G25100.3	cp, mt	324/36.26	212/23.78
	—	FeSOD2	—	AT5G51100.1	cp	—	305/34.66
	FeSOD3	FeSOD3	XM_010687302.1	AT5G23310.1	cp	262/30.25	263/30.35
	Cu/ZnSOD1	Cu-ZnSOD1	XM_010676650.1	AT1G08830.1	cyt, ecm, nuc	152/15.25	152/15.10
	Cu/ZnSOD2	Cu-ZnSOD2	XM_010690943.1	AT2G28190.1	cyt, cp, ecm	228/23.13	216/22.24
	Cu/ZnSOD3	Cu-ZnSOD3	XM_010695513.1	AT5G18100.1	cp, cyt, ecm, per, vac	157/15.81	164/16.94
	MnSOD1	MnSOD1	XM_010672327.1	AT3G10920.1	mt	230/25.58	231/25.44
	—	Fe-MnSOD	—	AT3G56350.1	mt	—	241/26.89
Peroxiredoxin (Prx) 2P-SH+H_2_O_2_→P-S-S-P+2H_2_O	1-Cys-Prx	1-Cys-Prx	XM_010669313.1	AT1G48130.1	cyt, nuc	219/24.41	216/24.08
	—	2-Cys-PrxA	—	AT3G11630.1	ecm, cp	—	266/29.09
	2-Cys-Prx	2-Cys-PrxB	XM_010685313.1	AT5G06290.1	ecm, cp, mt	271/ 29.74	273/29.77
	2-Cys-PrxIIF	2-Cys-PrxIIF	XM_010696978.1	AT3G06050.1	mt	174/18.66	201/21.44
	PrxQ	PrxQ	XM_010689254.1	AT3G26060.1	cp, plast	214/23.65	216/23.67
	—	PrxIIA	—	AT1G65990.1	cyt, nuc	—	553/62.65
	PrxIIB	PrxIIB	XM_010693075.1	AT1G65980.1	cp, cyt, pm	162/17.51	162 /17.42
	—	PrxIIC	—	AT1G65970.1	cyt	—	162/17.41
	—	PrxIID	—	AT1G60740.1	cyt, pm	—	162/17.47
	PrxIIE	PrxIIE	XM_010685588.1	AT3G52960.1	cp, pl, cw	227/24.07	234/24.68
Alternative oxidase (AOX) 2e^−^+2H^+^+O_2_→H_2_O	AOX1A	AOX1A	XM_010679200.1	AT3G22370.1	mt	353/39.62	354/39.98
	AOX1B	AOX1B	XP_010677502.1	AT3G22360.1	mt	102/11.19	325/37.43
	—	AOX1C	—	AT3G27620.1	mt	—	329/37.82
	—	AOX1D	—	AT1G32350.1	mt	—	318/36.20
	AOX2	AOX2	XM_010692188.1	AT5G64210.1	mt	329/37.69	353/40.09
	PTOX1	PTOX	XM_010688453.1	AT4G22260.1	cp	363/41.77	351/40.57
	PTOX2	—	XP_010686755.1	—	cp	596 /65.40	—
NADPH oxidase (RBOH) NADPH+e^−^+O_2_→NADP^−^+O^−^_2_+H^+^	—	RBOH A	—	AT5G07390.1	pm	—	902/102.94
	RBOH B	RBOH B	XM_010675737.1	AT1G09090.2	pm	887/100.94	843/96.39
	—	RBOH C	—	AT5G51060.1	pm	—	905/102.52
	—	RBOH D	—	AT5G47910.1	pm	—	921/ 103.91
	RBOH E	RBOH E	XM_010689256.1	AT1G19230.1	pm	945/ 106.04	934/105.55
	RBOH F	RBOH F	XM_010678094.1	AT1G64060.1	pm	947/107.72	944/108.42
	—	RBOH G	—	AT4G25090.1	pm	—	849/96.86
	RBOH H	RBOH H	XM_010687568.1	AT5G60010.1	pm	853/96.65	886/100.63
	—	RBOH I	—	AT4G11230.1	pm	—	941/106.95
	—	RBOH J	—	AT3G45810.1	pm	—	912/102.94
	RBOH K	—	XM_010696961.1	—	pm	797/ 91.11	—

### Transcriptional regulation of *Prx* genes

The sugar beet genome was searched for genes encoding peroxiredoxins (Dietz, 2011). While ten *Prx* genes were identified in Arabidopsis, only six were identified in sugar beet and were used to construct a phylogenetic tree (Fig. 4B). Sugar beet encodes only one cytosolic Prx, *PrxIIB*, while Arabidopsis encodes three and one homologous pseudogene *At-PrxIIA*. The sugar beet genome contains a single copy of mitochondrial *PrxIIF*, nuclear *1-CysPrx*, *2-CysPrx* and *PrxQ.* Among these thiol peroxidases, *1-CysPrx, PrxQ* and *PrxIIE* transcripts were lower in salt-treated plants at 3 h after up-salting (Fig. 5G–L). At the end of the experiments only transcripts encoding the two chloroplast Prxs, *2-CysPrx* and *PrxQ*, were increased in salt-stressed plants, while cytosolic *PrxIIB* transcript levels were decreased and all others were similar between both treatments. Relatedness was confirmed by sequence alignment (Supplementary Fig. S5). In order to complement these data, tissue distribution was analyzed (Supplementary Fig. S6) and revealed rather ubiquitous expression, with the exception of FeSOD1 and CuZnSOD2 that were scarcely expressed in seeds. Likewise PrxQ was absent from seeds and 1-CysPrx expression was very low in leaves (Supplementary Fig. S6).

### Transcript regulation of alternative and NADPH oxidases

**Fig. 6.**
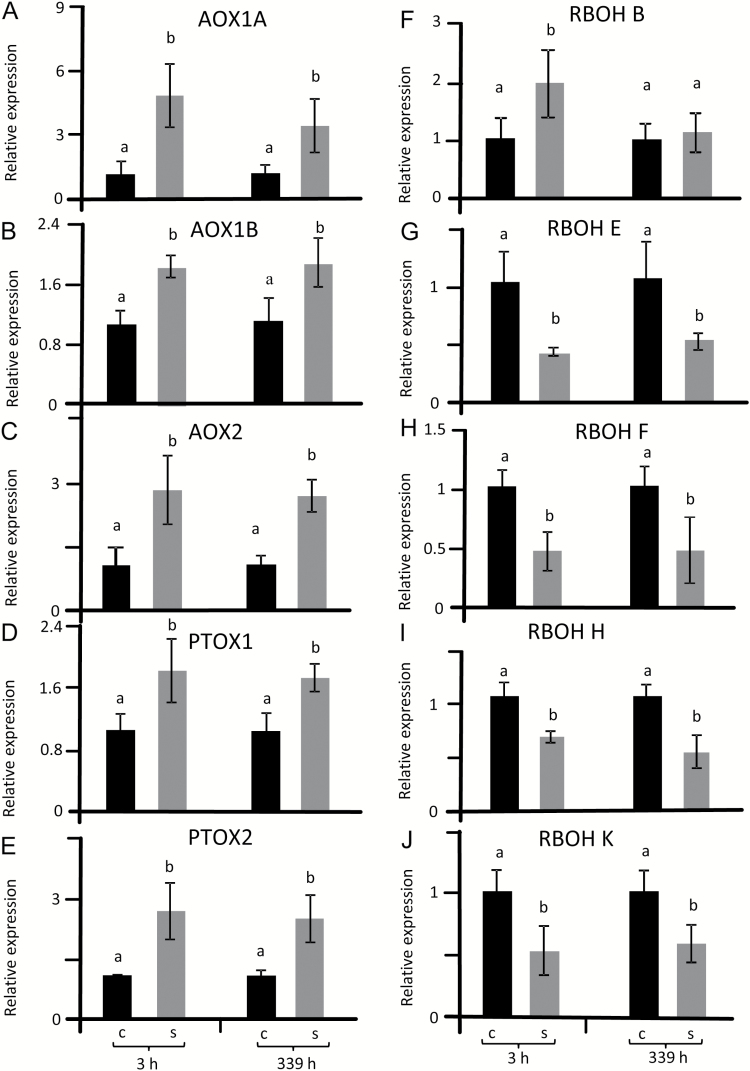
Transcript levels of AOX/PTOX and RBOH in sugar beet from stressed and non-stressed plants. Samples were taken at 3 h and 14 d after reaching a concentration of 300 mM NaCl as shown in Fig. 1. Transcript amounts were quantified by qPCR and calculated relative to actin levels. qPCR data represent the average of three independent experiments with two technical replicates each. The significant difference was calculated using Student’s *t*-test and labelled with different letters after analysis with Fisher LSD test, with *P*<0.05, using InfoStat statistical software. C, control; S, salinity.

To address the regulation of redox and ROS homeostasis, the sugar beet genome was searched for homologs of important safety valves, namely alternative oxidases (AOX) in mitochondria, plastid terminal oxidase (PTOX) as well as the ROS generation systems of NADPH oxidases, including the respiratory burst oxidase homologue RBOH. Phylogenetic analysis revealed three sequences in sugar beet related to Arabidopsis *AtAOX1* and *AtAOX2* sequences and two with similarity to *At-PTOX* (Fig. 4C, Table 1, Supplementary Figs S7, S8). Each of the *BvAOX* and *BvPTOX* trancripts was significantly up-regulated in salt-treated sugar beet at 3 h and 14 d (Fig. 6A–E). The search for *RBOH* genes in the sugar beet genome generated seven hits. Phylogenetic analysis revealed one homolog each for *AtRBOHA/B/C/D/G*-, *E*-, *F/I*, *H/J-like*, namely *BvRBOHB*, *BvRBOHE*, *BvRBOHF*, and *BvRBOHH* (Fig. 4D). One *BvRBOH* isoform was particularly different and named *BvRBOHK* (Fig. 4D, Supplementary Figs S7, S9). With the exception of *BvRBOHB* that was up-regulated 3 h after salt treatment, all other *BvRBOH* isoforms were significantly suppressed under salinity both at 3 h and 14 d (Fig. 6F–J).

### *Cis*-regulatory element analysis

The promoters of the above identified stress-responsive genes of sugar beet were investigated for the presence of *cis*-regulatory elements. This was carried out to gain some insight into gene regulation and plant signaling under stress conditions. The occurrence of special *cis*-elements appears to play important roles in the differential regulation of salinity-induced transcripts in *B. vulgaris* as compared with *A. thaliana*. Over-representation or exclusive occurrence in *B. vulgaris* was observed for GATA, WRKY, bHLH, TCP, DOF, ZF-HD, NF-YB, TALE, TBP, dehydrin and BES1 *cis*-elements (Supplementary Table S3–S5). Over-represented *cis*-elements vary within *B. vulgaris*, with GATA and WRKY more abundant in promotor regions of down-regulated transcripts and bHLH, TCP, dehydrin and BES1 more abundant in up-regulated transcripts (Supplementary Tables S3, S4).

### Antioxidant enzyme activities

**Fig. 7.**
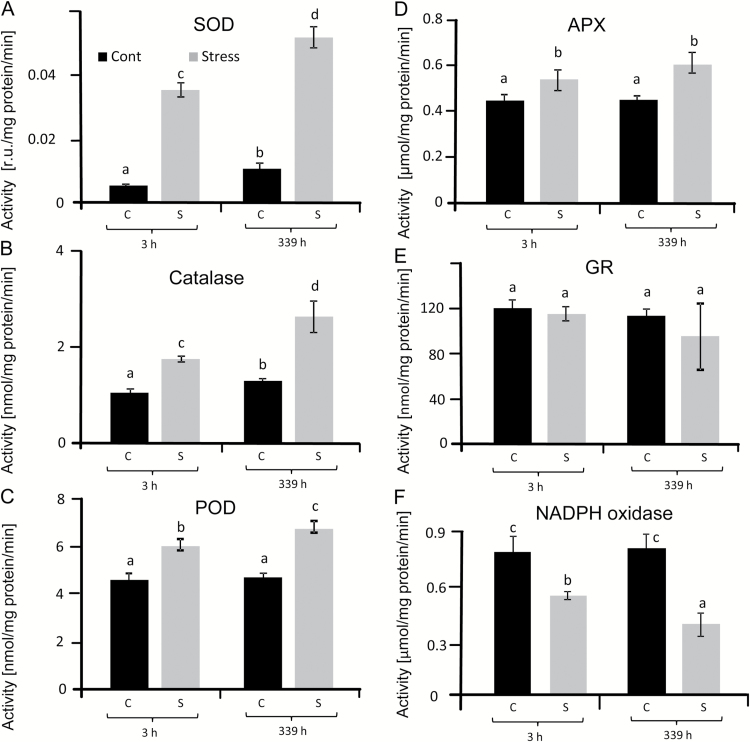
Activities of antioxidant enzymes in sugar beet leaves under salinity stress and control conditions: SOD (A), CAT (B), peroxidase (POD) (C), ascorbate peroxidase (APX) (D), glutathione reductase (GR) (E) and NADPH oxidase (RBOH) (F). Data are means ±SD of *n*=5 experiments with three measurements each. The significant difference was calculated using Student’s *t*-test and labelled with different letters after analysis with Fisher LSD test, with *P*<0.05, using InfoStat statistical software. C, control; S, salinity.

Activities of SOD, catalase, GR, APX and peroxidase (POD) were determined in leaves from salt-stressed and control plants in order to assess the state of the antioxidant systems (Fig. 7). Higher SOD activity was detected in stressed leaves, with the highest activity at 14 d with a 5-fold increase compared with controls (Fig. 7A). GR activity (Fig. 7E) was unaffected by saline growth conditions. However all other antioxidant enzyme activities were stimulated under salinity, with total APX activity showing the least increase, followed by total POD activity and catalase (Fig. 7B–D). Finally, NADPH oxidase activity was determined in plasma membrane-enriched membrane fractions of sugar beet leaves (Fig. 7F). Interestingly, NADPH oxidase was constant in control leaves at 3 h and 14 d, while it decreased by ~40% in salt-stressed plants at 3 h after up-salting up to 300 mM NaCl and by >60% at 14 d.

## Discussion

Sugar beet is able to grow in saline soils (Ghoulam *et al.*, 2002; Yang *et al.*, 2012; Yang *et al.*, 2013). In line with previous studies our results revealed efficient growth of sugar beet in medium with 300 mM NaCl. This was indicated by unchanged ΦPSII and maintained growth of ~50% of control conditions. Most notably, accumulated H_2_O_2_ levels were lower under salinity than control conditions. This response attracted our attention since salt stress usually stimulates ROS accumulation (Miller *et al.*, 2010). Concomitantly, levels of non-protein thiols, oxidized glutathione and ascorbate were decreased in salinized sugar beet. Sugar beet therefore appears to adjust the cellular redox milieu to a lower level of oxidative stress in high NaCl conditions than under control conditions. This regulatory mechanism contrasts the response of other plant species that display salt stress-induced enhancement of ROS accumulation, such as maize (AbdElgawad *et al.*, 2016), rice (Wutipraditkul *et al.*, 2015) and Arabidopsis (Ben Rejeb *et al.*, 2015).

The time-dependent increases in sodium and chloride content as well as osmotic potential (Supplementary Fig. S2) indicate the strong impact of salinity stress and precludes exclusion strategies in sugar beet. Maintenance of 50% photosynthetic CO_2_ fixation and unaltered ΦPSII demonstrates the high acclimation ability of sugar beet to strongly raised NaCl levels. The reduced growth rate quantitatively mirrored the reduced CO_2_ fixation rate. Efficient non-photochemical quenching may contribute to lowered ROS burden under salinity. Increased osmotic potential and transient ROS accumulation in halophyte roots are suggested to be instrumental in short-term activation of antioxidant defense, as well as in triggering expression of transcription factors important during long-term salinity (Ellouzi *et al.*, 2014). ROS accumulation was found in the glycophyte Arabidopsis during short- and long-term salt stress (Ellouzi *et al.*, 2011; Ellouzi *et al.*, 2014). Osmotic shock triggered by salinity stress may activate initial ROS production for subsequent activation of defense mechanisms (Choudhury *et al.*, 2013).

### Efficient response of SOD in salt-stressed sugar beet

Sugar beet contains homologs of each SOD form typically present in plants such as *A. thaliana* supporting the assumed conserved functions. High SOD activity is needed for rapid detoxification of O_2•_^−^, for example to minimize lipid peroxidation and peroxinitrate formation, if nitric oxide is formed concomitantly. Peroxynitrite (ONOO•^−^) is highly reactive with many cellular constituents and reacts with various amino acid residues in proteins, in particular with Phe, Trp, Tyr, His, Met and Cys residues (reviewed by Mock and Dietz, 2016). SOD, which is present in multiple subcellular compartments, is considered a first line of defense against ROS (Alscher *et al.*, 2002). With the exception of FeSOD1 transcript levels, which were down-regulated after 3 h and 339 h of salt stress, transcripts of CuZnSOD2, CuZnSOD3, CuZnSOD1, FeSOD3 and MnSOD1 were highly up-regulated under long-term salinity stress. CuSOD2 transcripts showed a small but significant increase after 3 h. As expected, based on the phylogenetic relatedness of *SOD* genes and the deduced amino acid sequences, *MnSOD* and *FeSOD* genes were highly related in sugar beet with sequence identity between both types of 93.6%. In contrast *CuZnSOD* genes have a very low sequence similarity with *FeSOD* and *MnSOD* genes. They separated into a unique cluster (Fig. 4a, Supplementary Fig. S4, Supplementary Table 2) and probably evolved separately in eukaryotes (Alscher *et al.*, 2002). Alscher *et al.* (2002) suggested that the spatial and temporal function of SOD isozymes is defined by their subcellular location and conditional expression regulated by upstream sequences in their promoters. The pattern of salt-induced transcript responses in sugar beet differed from that reported in Arabidopsis, where *CuSOD* genes were scarcely responsive while *FeSOD* genes responded the most (Mittler *et al.*, 2004). The focus of this study was directed towards acclimation to salinity and less to the immediate response to up-salting. Importantly, total SOD activity was up 7-fold at 3 h and 5-fold at 339 h (Fig. 7A). SOD activity determined during up-salting revealed first a parallel increase with H_2_O_2_ and then stabilized SOD activity with decreasing H_2_O_2_ at the end of the up-salting period (Fig. 1D). Up-regulation of SOD participates in adjusting the low ROS state. Produced H_2_O_2_ must be detoxified; this is facilitated by increased activities of catalase and APX (Fig. 7) and increased amounts of 2-CysPrx and PrxQ (Fig. 3). Stimulation of the ascorbate glutathione cycle is suggested to be an important mechanism of salinity tolerance (Stepien and Klobus, 2005).

### The role of peroxiredoxins in sugar beet

In higher plants, the minimum set of Prx is six isoforms: one plastidal (PrxIIE), one mitochondrial (PrxIIF), one cytosolic type II Prx (PrxIIB), one cytosolic/nuclear 1-CysPrx, one plastid 2-CysPrx and one plastidal PrxQ (Dietz *et al.*, 2006). Arabidopsis and rice express additional isoforms, ten and nine, respectively. Interestingly our search of the sugar beet genome conforms with the predicted minimum set of six Prx genes. The enzymatic activity of the different plant *Prx* isoforms has been well explored but the precise function within the redox regulatory network of the cell is still elusive. Functions as peroxidases, proximity peroxidases, ROS sensors, interaction partners and chaperones have all been proposed (König *et al.*, 2012). *2-Cys-PrxB* and *PrxQ* transcripts and protein levels were slightly increased during long-term salinity stress, while all other transcript levels decreased or were unaltered. The unresponsiveness of the single cytosolic *PrxIIB* gene contrasted results from Arabidopsis where *AtPrxIIB, C* and *D* showed strong responses to salinity and other stresses (Horling *et al.*, 2002; Horling *et al.*, 2003). This difference suggests that gene duplication in Arabidopsis allowed for diversification in stress response, while *BvPrxIIB* adopts a function more similar to housekeeping. These results indicate that 2-CysPrx contributes to the protection of chloroplast structures against oxidative damage by participating in detoxification processes (Baier and Dietz, 1999*a*,*b*). Furthermore, König *et al.* (2002) proposed that 2-Cys-Prx acts not only in the water–water cycle pathway for energy dissipation in photosynthesis but also in peroxide detoxification in plastids during the dark phase. *1-CysPrx* transcripts strongly but transiently decreased during salt stress. 1-CysPrx in Arabidopsis is highly abundant in seeds compared with root tissues but was not expressed in leaf tissues or only in some cells like petiole junctions (Stacy *et al.*, 1996; Haslekas *et al.*, 1998; Stacy *et al.*, 1999). 1-CysPrx in *A. thaliana* and *B. vulgaris* comprise 216 and 219 amino acids, respectively, possess a C-terminal extension with a putative nuclear signal, and as shown for Arabidopsis is localized in the nucleus and cytosol (Rouhier and Jacquot, 2002).

### The *AOX* gene family and their transcript response to salinity

Alternative oxidases function in the dissipation of reducing power in energetic electron transport of both mitochondria (AOX) and chloroplasts (PTOX). In the sugar beet genome, three *AOX* genes were identified that were highly similar to *AtAOX1* and *AtAOX2* groups in *A. thaliana* and therefore named *BvAOX1A*, *BvAOX1B* and *BvAOX2*. Two genes coding for proteins with high homology to PTOX were identified in sugar beet and named *BvPTOX1* and *BvPTOX2*. Efficient and coordinated up-regulation of AOX and PTOX may represent a specific feature of sugar beet during salinity stress tolerance because transcripts of all four AOX and AOX-like genes as well as PTOX were up-regulated both at 3 h and 339 h of salt stress. Up-regulation of PTOX and AOX probably stabilize the photosynthetic quantum yield of PSII under salinity despite inhibited photosynthesis. Overexpression of *AtAOX1a* lowers ROS formation in leaves (Smith *et al.*, 2009). This result is in line with the hypothesis that AOX participates in cell reprogramming under salinity stress. Activation of AOX limits ROS release from the mitochondrial respiratory chain. This activation is achieved in Arabidopsis by transcriptional control and by post-translational mechanisms (Rhoads *et al.*, 1998; Smith *et al.*, 2009). If AOX-dependent dissipation of excess reducing power is absent, ROS accumulates and can diffuse to other cell compartments (reviewed by Dietz *et al.*, 2016). Thus AOX contributes to mitigating oxidative stress under conditions of high salinity stress (Hanqing *et al.*, 2010). Moreover, genetic enhancement of AOX expression decreases ROS levels in transgenic tobacco (Maxwell *et al.*, 1999). Also, the abundance of PTOX positively correlates with salinity levels (Ivanov *et al.*, 2012; Nawrocki *et al.*, 2015). From these findings we conclude that transcriptional up-regulation of AOX and PTOX participates in the suppression of ROS accumulation in salt-stressed sugar beet.

### The *RBOH* gene family and their regulation

Sugar beet expresses five *RBOH* and *RBOH*-like genes compared to ten in Arabidopsis, where *AtRBOHD* and *AtRBOHF* preferentially accumulate upon salt stress (Ma *et al.*, 2012). Arabidopsis single mutants devoid of *AtrbohD* or *AtrbohF* are indistinguishable from wild type if stressed with 300 mM NaCl. Conversely, the double mutant *AtrbohD/F* reveals reduced viability in 300 mM NaCl administered for 14 d and simultaneously accumulates less H_2_O_2_ (Ma *et al.*, 2012). Interestingly in sugar beet viability remained at 100% under salt stress despite the strong decrease in RBOH activity. Ben Rejeb *et al.* (2015) assigned a crucial role to RBOHs in salinity-induced regulation of the antioxidant defense in Arabidopsis under short-term treatments. The salinity-induced activation of endosomal RBOH is suppressed in the phosphatidylinositol-3-kinase mutant *pi3k* (Leshem *et al.*, 2007) resulting in less oxidative stress. Post-translational control of RBOH by cellular Ca^2+^, ROS and phosphorylation networks participates in the early responses to salinity, particularly in glycophytes but possibly also in halophytes (Kurusu *et al.*, 2015). Moreover, *BvRBOHB* levels doubled at 3 h in contrast to all other RBOH transcripts that were down-regulated both at 3 h and 339 h. Thus *BvRBOHB* may be involved in early acclimation responses to ionic and osmotic stress, similar to *AtRBOHD* and *AtRBOHF* in Arabidopsis (Ma *et al.*, 2012).

### Tight regulation of redox and ROS in salt-stressed sugar beet

All the data from this study lead to a consistent response pattern of sugar beet under salt stress, which ensures low ROS accumulation and maintains metabolism and growth. Up-regulation of AOX and PTOX facilitates the dissipation of excessive reducing power accumulating in stressed plants. The efficiency of this regulation is indicated by the unchanged and high ΦPSII in the growth of light-exposed plants, revealing an avoidance of over-reduction. Over-reduced electron transport chains are a prime source for the generation of superoxide, singlet oxygen and other ROS (Oelze *et al.*, 2008). Likewise the activity of RBOH as a ROS generator system is strongly suppressed in short- and long-term salt-treated sugar beet. Up-regulation of SOD, APX, POD, catalase and some Prxs assists in lowering steady state ROS levels. Here the described efficient regulation of the ROS network in sugar beet provides a solid basis for futher investigating the underlying regulation at the level of signaling and transcription factors, including *cis*-elements and hormones such as abscisic acid (ABA).

ABA-dependent regulation via dehydrin in *B. vulgaris* may be an additional regulatory mechanism compared with *A. thaliana*, which coordinates up-regulation of antioxidative enzymes and lowers H_2_O_2_ levels under salt stress as compared with controls (Table 3). The cooperative action of *cis*-elements COR15 (dehydrin) and the promoter configuration are crucial for the regulation of ABA-induced (Busk and Pagès, 1998) and drought-regulated gene expression (Baker *et al.*, 1994). Under salinity dehydrin1 overexpressing plants maintain lower H_2_O_2_ levels than wild type plants (Kumar *et al.*, 2014). Dehydrin may constitute part of a general molecular mechanism used by all land plants to protect them from injury during cellular dehydration under salinity and osmotic stress (Saavedra *et al.*, 2006).

BES1 is only present in up-regulated transcripts of antioxidant enzymes and therefore might function as a positive regulator in *B. vulgaris* leading to higher antioxidant defense and higher salt tolerance (Table 3). The BES1 *cis*-element family binds plant-specific transcription factors that cooperate with the bHLH transcription factor BIM1 to regulate brassinosteroid-induced abiotic stress responsive gene expression (Yin *et al.*, 2005). BES1 and AtMYB30 function cooperatively to promote brassinosteroid target gene expression through a mechanism by which AtMYB30, a direct target of BES1, amplifies brassinosteroid signaling by helping BES1 activate downstream target genes (Li *et al.*, 2009). It will be of interest to compare the genome-wide distribution and function of these elements in sugar beet and glycophytes in the future.

## Supplementary Material

Supplementary Data

## Abbreviations:

ΦPSII: quantum yield of photosystem II
2-CysPrx: 2-cysteine peroxiredoxin
AA: ascorbate
ABA: abscisic acid
APX: ascorbate peroxidase
AOX: mitochondrial alternative oxidase
CO_2_: carbon dioxide
DHA: dehydroascorbate
DTNB: 5,5’-dithiobis(2-nitrobenzoate)
GPX: glutathione peroxidases
GR: glutathione reductase
GSH: glutathione
GSSG: oxidized glutathione
H_2_O_2_: hydrogen peroxide
MDA: malondialdehyde
ML: maximum likelihood
NaCl: sodium chloride
NPT: non-protein thiol
O_2_•^−^: superoxide
ONOO•^−^: peroxynitrite
PEG: polyethylene glycol
POD: peroxidase
Prx: peroxiredoxins
PrxQ: chloroplast peroxiredoxin Q
PSII: photosystem II
PTOX: plastid terminal oxidase
RBOH: respiratory burst oxidase homolog
ROS: reactive oxygen species
SOD: superoxide dismutase
TBA: thiobarbituric acid
TBST: Tris-buffered saline with 0.1% Tween 20
TCA: trichloroacetic acid
XTT: Na 3′-[1-[phenylaminocarbonyl]-3,4-tetrazolium]-bis(4-methoxy-6-nitro)benzenesulfonic acid.
