# Supplementary material for: Redox and Reactive Oxygen Species Network in Acclimation for Salinity Tolerance in Sugar Beet
Source: J Exp Bot. 2017 Feb 18;68(5):1283–98. doi: 10.1093/jxb/erx019 (PMC5441856; doi:10.1093/jxb/erx019)
Supplement: Supplementary Data [file erx019_Supplementary_Data.zip › supplementary_figures_S1_S9.pdf]

Supplementary material for Hossain et al.

Suppl. Fig. 1

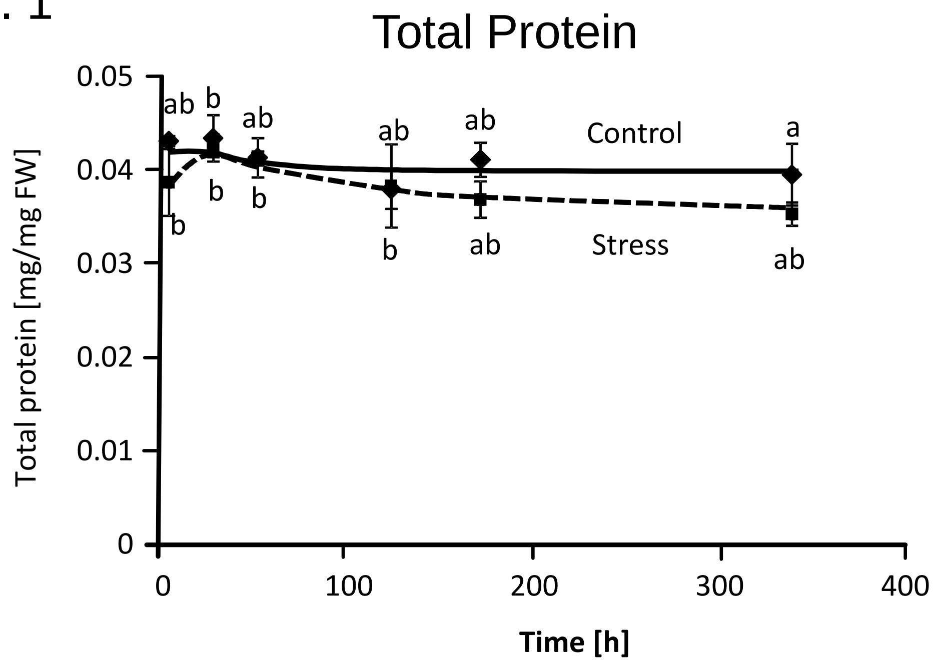

Supplementary Figure 1: Total protein contents of sugarbeet leaves in salt stressed and control plants at different time point after treatment with 300 mM NaCl. Data are given as means ± SD; different letters mark significance groups by using Fisher LSD test and t-test, $p<0.05$.

Suppl. Fig. 2

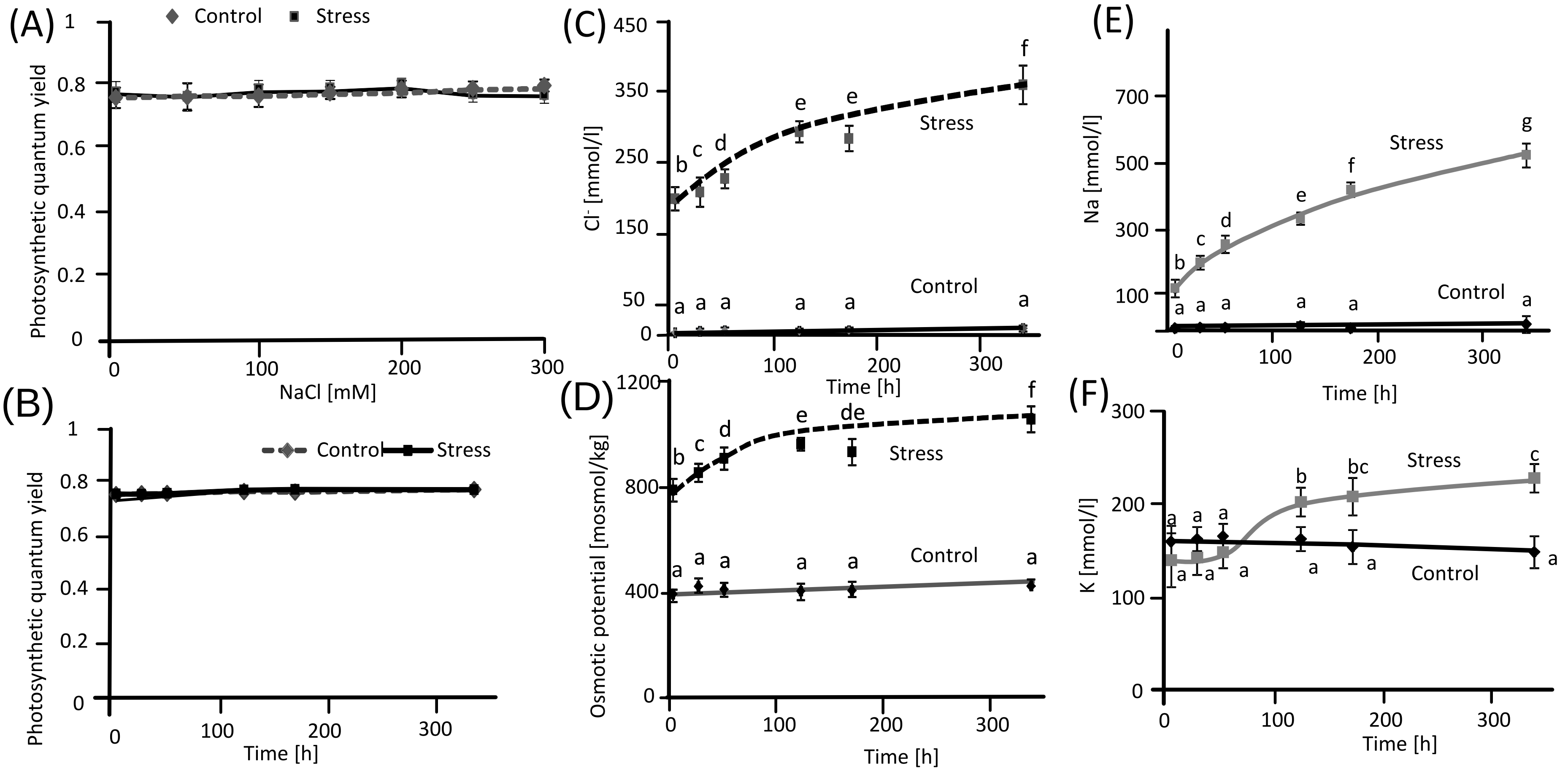

Supplementary Figure 2: Photosynthetic quantum yield (ΦPSII), osmotic potential, sodium, potassium and chloride contents of sugarbeet leaves under control and salt stress conditions. (A) Steady-state ΦPSII of leaves from control and stressed plants during the salting up experiment from 0 to 300 mM NaCl. (B) Steady-state ΦPSII during the subsequent 14d after salting up. (C) Chloride contents of NaCl-treated and unstressed sugarbeet leaves. (D) Osmotic potential determined by freezing point depression in salt-stressed and control leaf extracts during 14d. (E) Sodium contents of NaCl-treated and unstressed sugarbeet leaves. (F) Potassium contents of NaCl-treated and unstressed sugarbeet leaves. Data are means ±SD of n=5 experiments with five measurements each. Data groups of significant difference are labelled with different letters using Fisher LSD test ($p \leq 0.05$, Student's t test).

Suppl. Fig. 3

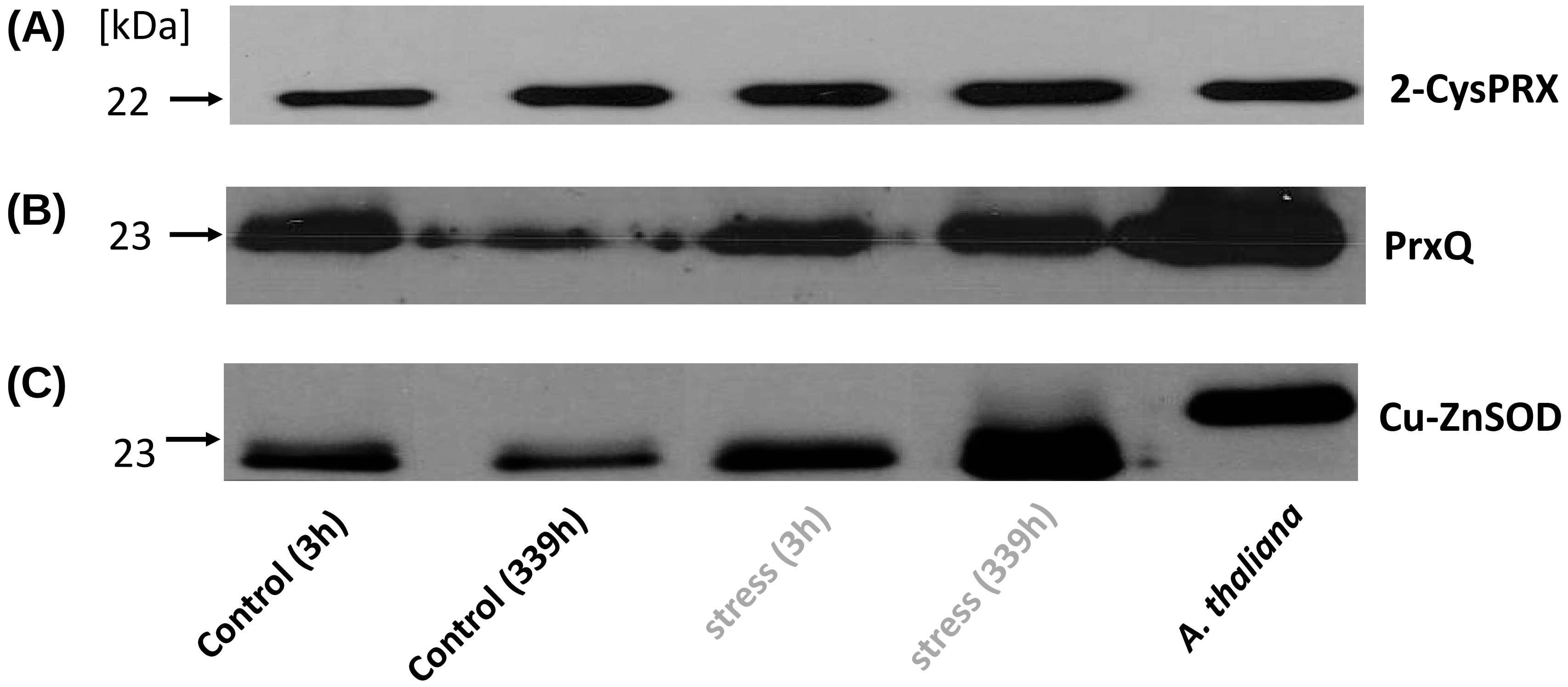

Supplementary Figure 3: Quantification of 2-CysPRX, PRXQ and CuZnSOD by Western blot analysis of salt stressed and control sugarbeet. (a) Total leaf protein was separated by reducing SDS PAGE, and 2-CysPRX detected with 2-CP-antibody. (b) Western blot-based quantification of PRXQ. (c) Western blot-based quantification of Cu-ZnSOD.

Suppl. Fig. 4

Supplementary Figure 4: Alignment of the deduced amino acid sequences of SODs in *Beta vulgaris* and *Arabidopsis thaliana* according to GenBank database. (*) indicate full conservation among all sequences, (:) highly conservative exchanges and (.) conservative exchanges of amino acids.

```
At-Cu-ZnSOD1      ------------------------------------MAKGVAVLNSSEGVTGTI-----F 19
Bv-Cu-ZnSOD1      ------------------------------------MGKAVVVLNSSEGVTGTI-----Y 19
At-Cu-ZnSOD3      ------------------------------MEAPRGNLRAVALIAGDNNVRGCL-----Q 25
Bv-Cu-ZnSOD3      ------------------------------MGT---TVKAVAVIAGSNNVKGSL-----H 22
At-Cu-ZnSOD2      GVSLNNNNL-----HRLQSVSFAVKAPSKALTVVSAAKKAVAVLKGTSDVEGVV-----T 50
Bv-Cu-ZnSOD2      SSTSSFNGLSFKLSSQIQSLSVSSSVAPKPLTIVAASKKAVAVLKGTSNVEGVV-----T 55
                                                        :.*.:: . ..* * :

At-Cu-ZnSOD1      FTQEGDGVTTVSGTVSGLKPG----LHGFHVHALGDT--TNGCMSTGPHFNPDGKTHGAP 73
Bv-Cu-ZnSOD1      FTQEGDGPTTVTGNISGLKPG----LHGFHVHALGDT--TNGCMSTGPHYNPAGKEHGAP 73
At-Cu-ZnSOD3      FVQDISGTTHVTGKISGLSPG----FHGFHIHSFGDT--TNGCISTGPHFNPLNRVHGPP 79
Bv-Cu-ZnSOD3      FIQHSTGITHVKGKITGLTPG----LHGFHIHALGDT--TNGCNSTGPHFNPMKKDHGAP 76
At-Cu-ZnSOD2      LTQDDSGPTTVNVRITGLTPG----PHGFHLHEFGDT--TNGCISTGPHFNPNNMTHGAP 104
Bv-Cu-ZnSOD2      LTQEDDGPTTVNVRITGLTPG----KHGFHLHEFGDT--TNGCISTGPHFNPSSMTHGAP 109
                  : *.  * * *.  ::**.**     ****:* :***  **** *****:**    **.*

At-Cu-ZnSOD1      EDANRHAGDLGNITVGD--------------------------DGTATFTITDCQIPLTG 107
Bv-Cu-ZnSOD1      EDEVRHAGDLGNVTVGD--------------------------DGTATFTIIDSQIPLCG 107
At-Cu-ZnSOD3      NEEERHAGDLGNILAGS--------------------------NGVAEILIKDKHIPLSG 113
Bv-Cu-ZnSOD3      SDTERHVGDLGNIVAGS--------------------------DGVAEVSISDSQISLSG 110
At-Cu-ZnSOD2      EDECRHAGDLGNINANA--------------------------DGVAETTIVDNQIPLTG 138
Bv-Cu-ZnSOD2      EDEIRHAGDLGNIVANT--------------------------DGVAEATIIDNQIPLSG 143
                  .:  **.*****: ..                           :*.*   * * :*.* *

At-Cu-ZnSOD1      PN----SIVGRAVVVHADPDDLGKGGHELSLATGNAGGRVACGIIGLQG 152
Bv-Cu-ZnSOD1      PN----SVVGRAVVVHADPDDLGRGGHELSKTTGNAGGRVACGVIGLQG 152
At-Cu-ZnSOD3      QY----SILGRAVVVHADPDDLGKGGHKLSKSTGNAGSRVGCGIIGLQS 158
Bv-Cu-ZnSOD3      QH----SILGRAVVVHADPDDLGRGGHELSKATGNAGARVGCGVIGLQS 155
At-Cu-ZnSOD2      PN----SVVGRAFVVHELKDDLGKGGHELSLTTGNAGGRLACGVIGLTP 183
Bv-Cu-ZnSOD2      AN----SVVGRALVVHELEDDLGKGGHELSLTTGNAGGRLACGVVGLTP 188
                        *::***.***   ****:***:** :*****.*:.**::**

At-FeSOD1         AASSAVTANYVLKPPPFALDALEPHMSKQTLEFHWGKHHRAYVDNLKKQVLGTE-LEGKP 59
Bv-FeSOD1         AGAAVVTAKFDLKPPPYPFDALEPHMSKQTFDYHWGKHHRAYVDNLNKQIVGTE-LDGLP 59
At-FeSOD2         AVSGVITAGFELKPPPYPLDALEPHMSRETLDYHWGKHHKTYVENLNKQILGTD-LDALS 59
At-FeSOD3         RGGLKVEAYYGLKTPPYPLDALEPYMSRRTLEVHWGKHHRGYVDNLNKQLGKDDRLYGYT 60
Bv-FeSOD3         KRRPGVVAYYGLKEPPYNLDALEPYMSQKTLESHWGEYHRGYLENLNRHLSRSDILYGYT 60
                       : * : ** **: :*****:**:.*:: ***::*: *::**::::   : * . .

At-FeSOD1         LEHIIHSTYNNGDLLPAFNNAAQAWNHEFFWESMKP---GGGGKPSGELLALLERDFTSY 116
Bv-FeSOD1         LEEVVRITYNKGDVLPSFNNAAQAWNHEFFWESMKP---SGGGKPSGELLAQIEKDFGSF 116
At-FeSOD2         LEEVVLLSYNKGNMLPAFNNAAQAWNHEFFWESIQP---GGGGKPTGELLRLIERDFGSF 116
At-FeSOD3         MEELIKATYNNGNPLPEFNNAAQVYNHDFFWESMQP---GGGDTPQKGVLEQIDKDFGSF 117
Bv-FeSOD3         MDDLVKVTYNYGNPLPEFNDAAQVWNHDFFWESMQP---GGGESPTLGLLQQIEKDFGSF 117
                  ::.::  :** *: ** **:***.:**:*****::*   .** .*   :*  :::** *:

At-FeSOD1         EKFYEEFNAAAATQFGAGWAWLAYSN-----------------EKLKVVKTPNAVNPLVL 159
Bv-FeSOD1         EAFVTEFKTAGATQFGSGWAWLVYKTNKLDVGNAVNPKPSEDDKKLAVVKSPNAVNPLVW 176
At-FeSOD2         EEFLERFKSAAASNFGSGWTWLAYKANRLDVANAVNPLPKEEDKKLVIVKTPNAVNPLVW 176
At-FeSOD3         TNFREKFTNAALTQFGSGWVWLVLKR---------------EERRLEVVKTSNAINPLVW 162
Bv-FeSOD3         TDFKEKFIEAAMTHFGSGWVWLVLKR---------------KEKQLAVVKTSNAVTPIVW 162
                    *  .*  *. ::**:**.**. .                  .:* :**:.**:.*:*

At-FeSOD1         GS---FPLLTIDVWEHAYYLDFQNRRPDYIKTFMTNLVSWEAVSARLEA 205
Bv-FeSOD1         DY---YPLLTVDVWEHAYYLDFQNRRPDYISIFMENLVSWDAVSVRYEA 222
At-FeSOD2         DY---SPLLTIDTWEHAYYLDFENRRAEYINTFMEKLVSWETVSTRLES 222
At-FeSOD3         DD---IPIICVDVWEHSYYLDYKNDRAKYINTFLNHLVSWNAAMSRMAR 208
Bv-FeSOD3         DD---IPIICLDLWEHAYYLDYKNDRAKYVNVFMNHLVSWHSATARLAR 208
                  .     *:: :* ***:****::* *..*:. *: :****.:.  *

At-MnSOD1         LLRIRGIQTFTLPDLPYDYGALEPAISGEIMQIHHQKHHQAYVTNYNNALEQLD----QA 56
Bv-MnSOD1         LLQCRSLQTFSLPDLPYDYGALQPAISGEIMQIHHQKHHQTYITNYNKALEQLD----DA 56
At-Fe-MnSOD       EPCLESMKTASLPDLPYAYDALEPAISEEIMRLHHQKHHQTYVTQYNKALNSLR----SA 56
                      ..::* :****** *.**:**** ***::*******:*:*:**:**:.*     .*

At-MnSOD1         VNKGDASTVVKLQSAIKFNGGGHVN-HSIFWKNLAPSSEGGGEPPKGSLGSAIDAHFGSL 115
Bv-MnSOD1         IAKGDASSVVKLQSAIKFNGGGHVN-HSIFWKNLAPINEGGGEPPKSSLGWAIDSNFGSL 115
At-Fe-MnSOD       MADGDHSSVVKLQSLIKFNGGGHVN-HAIFWKNLAPVHEGGGKPPHDPLASAIDAHFGSL 115
                  : .** *:****** ********** *:********  ****:**:..*. ***::****

At-MnSOD1         EGLVKKMSAEGAAVQGSGWVWLGLDK----------------ELKKLVVDTTANQDPLVT 159
Bv-MnSOD1         EALIQKMNAEGAAVQGSGWVWLGLDT----------------QSKKLVVETTPNQDPLVT 159
At-Fe-MnSOD       EGLIQKMNAEGAAVQGSGWVWFGLDR----------------ELKRLVVETTANQDPLVT 159
                  *.*::**.*************:***                 : *:***:**.*******

At-MnSOD1         KGGSLVPLVGIDVWEHAYYLQYKNVRPEYLKNVWK-VINWKYASEVYEK 207
Bv-MnSOD1         KGPSLVPILGIDVWEHAYYLQYKNVRPDYLKNIWK-VINWKYASEVYEK 207
At-Fe-MnSOD       KGSHLVPLIGIDVWEHAYYPQYKNARAEYLKNIWT-VINWKYAADVFEK 207
                  **  ***::********** ****.*.:****:*. *******::*:**
```

Suppl. Fig. 5

```
At-2-Cys-PrxA     --MASVASS--TTLISSPSSRVFPAKS--SLSSPSVSFLRTLSSPSASAS-LRSGFARRS 53
At-2-Cys-PrxB     MSMASIASSSSTTLLSS-SRVLLPSKS--SLLSPTVSFPRIIPSSSASSSSLCSGFSSLG 57
Bv-2-Cys-Prx      --MASAASS--SAILSPNPTRAFAAKAHAPMAVAVKPFSQTLNLSSNFNG-IRKSFQSPL 55
At-1-Cys-Prx      ------------------------------------------------------------
Bv-1-Cys-Prx      ------------------------------------------------------------

At-2-Cys-PrxA     SLS----STSRRSFAVKAQADDLPLVGNKAPDFEAEAVFDQEFIKVKLSDYIGKKYVILF 109
At-2-Cys-PrxB     SLTTNR-SASRRNFAVKAQADDLPLVGNKAPDFEAEAVFDQEFIKVKLSEYIGKKYVILF 116
Bv-2-Cys-Prx      SLSSRRTQSSKRSFVVRASS-ELPLVGNTAPDFEAEAVFDQEFINVKLSDYKGKKYVILF 114
At-1-Cys-Prx      ---------------MPGIT-----LGDTVPNLEVET----THDKFKLHDYFANSWTVLF 36
Bv-1-Cys-Prx      ---------------MPGIT-----IGDTVPNLEVET----TQGRFKLHDYFADSWTILF 36
                                 : . :     :*:..*::*.*:       ..** :* ...:.:**

At-2-Cys-PrxA     FYPLDFTFVCPTEIT-AFSDRHSEFEKLNTEVLG-VSVDSVFSHLAWVQTDRKSGGLGDL 167
At-2-Cys-PrxB     FYPLDFTFVCPTEIT-AFSDRYEEFEKLNTEVLG-VSVDSVFSHLAWVQTDRKSGGLGDL 174
Bv-2-Cys-Prx      FYPLDFTFVCPTEIT-AFSDRHGEFEKLNTEVLG-VSVDSVFSHLAWVQTDRKSGGLGDL 172
At-1-Cys-Prx      SHPGDFTPVCTTELG-AMAKYAHEFDKRGVKLLG-LSCDDVQSHKDWIKDIEAFNHGSKV 94
Bv-1-Cys-Prx      SHPGDFTPVCTTELG-MMAAYADEFSKRGVKLLG-LSCDDIASHNEWIKDVEAYSGGHKV 94
                   :* *** **.**:   ::    **.* ..::** :* *.: **  *::  .  .   .:

At-2-Cys-PrxA     NYPLISDVTKSISKSFGVLIHDQ-GIAL-----RGLFIIDKEGVIQHSTINNLG------ 215
At-2-Cys-PrxB     NYPLVSDITKSISKSFGVLIPDQ-GIAL-----RGLFIIDKEGVIQHSTINNLG------ 222
Bv-2-Cys-Prx      KYPLVSDVTKSISKAYNVLIPDQ-GIAL-----RGLFIIDKEGVIQHSTINNLA------ 220
At-1-Cys-Prx      NYPIIADPNKEIIPQLNMIDPIENGP------SRALHIVGPDSKIKLSFLYPST------ 142
Bv-1-Cys-Prx      KYPIIADPNREIIKQLNMVDPDEKGPNGQQLPSRALHIVGPNKMVKLSFLYPAT------ 148
                  :**:::* .:.*    .::   : *        *.*.*:. :  :: * :

At-2-Cys-PrxA     ----IGRSVDETMRTLQALQYIQENPDEVCPAG-WKPGEKSMKP---DPKLSKEYFSAI- 266
At-2-Cys-PrxB     ----IGRSVDETMRTLQALQYVQENPDEVCPAG-WKPGEKSMKP---DPKLSKEYFSAI- 273
Bv-2-Cys-Prx      ----IGRSVDETLRTLQALQYVQENPDEVCPAG-WKPGEKSMKP---DPKLSKEYFAAI- 271
At-1-Cys-Prx      ----TGRNMDEVLRALDSLLMASKHNNKIATPVNWKPDQPVVISPAVSDEEAKKMFPQGF 198
Bv-1-Cys-Prx      ----TGRNMDEVLRVVDSLQKAAKH--KVATPVNWKPNEKVVISPSVNNDQAKEMFPQGF 202
                       **.:**.:*.:::*    ::  ::...  ***.:  : .   . . :*: *.
```

```
At-PrexIIC        ----------------MAPITVGDVVPDGTISFFDE---NDQLQTVSVHSIAAGKKVILF 41
At-PrexIID        ----------------MAPITVGDVVPDGTISFFDE---NDQLQTVSVHSIAAGKKVILF 41
At-PrxIIA         ----------------MAPIDVGDFVPDGSISFFDD---DDQLQTVSVHSLAAGKKVILF 41
At-PrexIIB        ----------------MAPIAVGDVVPDGTISFFDE---NDQLQTASVHSLAAGKKVILF 41
Bv-PrxIIB         ----------------MAPLAVGDVIPDGNLKYFDE---NNQSQDVSIHSLSAGKIVVIF 41
At-PrxIIE         TNSASATTRSFATTPVTASISVGDKLPDSTLSYLDPS--TGDVKTVTVSSLTAGKKTILF 111
Bv-PrxIIE         KKPLHFST-TTTTPKIHASISVGSKLPDSTLSFLSP---TNDVETVTISDLTSSKKTILF 103
                                   *.: **. :**..:.::.     .: : .:: .:::.* .::*

At-PrexIIC        GVPGAFTPTCSMSHVPGFIGKAEELKSKGIDEIICFSVNDPFVMKAWGK-TYPEN----K 96
At-PrexIID        GVPGAFTPTCSMSHVPGFIGKAEELKSKGIDEIICFSVNDPFVMKAWGK-TYQEN----K 96
At-PrxIIA         GVPGAFPPTCSMNHVNGFIEKAEELKSNGVDEIICLSGDDPFMITACS-----EN----K 92
At-PrexIIB        GVPGAFTPTCSMKHVPGFIEKAEELKSKGVDEIICFSVNDPFVMKAWGK-TYPEN----K 96
Bv-PrxIIB         AVPGAFTPTCSQKHVPGFIEKAEELKSKGVAEILCISVNDQFVMKEWAK-TYPDN----K 96
At-PrxIIE         AVPGAFTPTCSQKHVPGFVSKAGELRSKGIDVIACISVNDAFVMEAWRKDLGIN-----D 166
Bv-PrxIIE         AVPGAFTPTCSQKHLPGFVAKAAELKSKGVDIIACISVNDAFVMKAWKENLNIEG----D 159
                  .*****.**** .*: **: ** **:*:*:  * *:* :* *::         :     .

At-PrexIIC        HVKFVADGSGEYTHLLGLELDLKDK--GLGIRSRRFALLLDNLKVTVANVESGG------ 148
At-PrexIID        HVKFVADGSGEYTHLLGLELDLKDK--GLGIRSRRFALLLDNLKVTVANVENGG------ 148
At-PrxIIA         HVKFVEDGSGEYIQLLGLELEVKDK--GLGVRSRGFALLLDNLKVIVVNVGSGGDCSLFQ 150
At-PrexIIB        HVKFVADGSGEYTHLLGLELDLKDK--GLGVRSRRFALLLDDLKVTVANVESGG------ 148
Bv-PrxIIB         HIKFLADGSGAYTRALGLELDLSDK--GLGIRSRRYALLVDDLKVKVANIESGG------ 148
At-PrxIIE         EVMLLSDGNGEFTGKLGVELDLRDKPVGLGVRSRRYAILADDGVVKVLNLEEGG------ 220
Bv-PrxIIE         DVLLLSDGNCDFTRAIGAELDLSDKPVGLGVRSKRYSMLVEDGVVKVINMEDGG------ 213
                  .: :: **.  :   :* **:: **  ***:**: :::* ::  * * *: .**

At-PrexIIC        --EFTVSSAEDILKAL-------------------------------------------- 162
At-PrexIID        --EFTVSSAEDILKAL-------------------------------------------- 162
At-PrxIIA         LMKMTTTTMSNLPTDLLEEIISRVPRKYMRAVRLTCKRWNGMFKSQSFTKMHIGKEEAAT 210
At-PrexIIB        --EFTVSSADDILKAL-------------------------------------------- 162
Bv-PrxIIB         --EFSVSSADEILKAL-------------------------------------------- 162
At-PrxIIE         --AFTNSSAEDMLKAL-------------------------------------------- 234
Bv-PrxIIE         --AFTSSGADDMLKVL-------------------------------------------- 227
                     :: :  .:: . *
```

```
At-PrxQ           --MAASSSS--FTLCNHTTLRTLPLRK----TLVTKTQ-FSVPTKSSESNFFGSTLTHSS 51
Bv-PrxQ           --MATLS------LPKHSLPFTLPSQT----PKFHSSQNLSIISKSSQSQFYGLKFSHSS 48
                    **: *      * :*:   ***  :.    . . .:* :*: :***:*:*:* .::***

At-PrxQ           YIS-PVSSSSLKGLIFAKVN-----KGQAAPDFTLK---DQNGKPVSLKKYKGK-PVVLY 101
Bv-PrxQ           SVSSPSTSRSFKAAIVAKIT-----EGSMAPAFTLK---DQDGKNVSLTKFKGK-PVVVY 99
                   :* * :* *:*. *.**:.     :*. ** ****   **:** ***.*:*** ***:*

At-PrxQ           FYPADETPGCTKQAC-AFRDSYEKFKKAGAEVIG-ISGDDSASHKAFASKYK-------L 152
Bv-PrxQ           FYPADETPGCTKQAC-AFRDSYEKFKKAGAEVIG-ISGDDSSSHKSFKQKYK-------L 150
                  *************** ****************** ******:***:* .***       *

At-PrxQ           PYTLLSDEGNKVRKDWGVPGDLFGALPG-----RQTYVLDKNGVVQLIYNNQFQ------ 201
Bv-PrxQ           PFTLLSDEGNKVRKDWGVPSDLFGALPG-----RQTYVLDRNGVVRLVYNNQFQ------ 199
                  *:*****************.********     *******:****:*:******

At-PrxQ           ----PEKHIDETLKFLKAA----------------------------------------- 216
Bv-PrxQ           ----PEKHIDETLKFLQSL----------------------------------------- 214
                      ************::
```

```
Bv-PrxIIF         ---------------------------------------MASSMVIKR-SSVLKSLTGGL 20
At-PrxIIF         ---------------------------------------MAMSILKLRNLSALRSAANSA 21
                                                         ** *::  *  *.*:* :..

Bv-PrxIIF         RVAAS-RAYASVAVGSDIVSAAPDISLQKARTYDEGV--SSKFSTTPLHDIFKGKKVVIF 77
At-PrxIIF         RIGVSSRGFSKLAEGTDITSAAPGVSLQKARSWDEGV--SSKFSTTPLSDIFKGKKVVIF 79
                  *:..* *.::.:* *:**.****.:******::****  ********* ***********

Bv-PrxIIF         GLPGAFTGVCSAQHVPSYKTNIDKLKAKGVDSIVCVSVNDPYVMHGWAEKLEAK-----D 132
At-PrxIIF         GLPGAYTGVCSQQHVPSYKSHIDKFKAKGIDSVICVSVNDPFAINGWAEKLGAK-----D 134
                  *****:***** *******::***:****:**::*******:.::****** **     *

Bv-PrxIIF         AIEFYGDFDASFHKSLDLTIDLSAA--LLGTRSHRWSAYVEDSKIKVLNVEKAP------ 184
At-PrxIIF         AIEFYGDFDGKFHKSLGLDKDLSAA--LLGPRSERWSAYVEDGKVKAVNVEEAP------ 186
                  *********..*****.*  *****  ***.**.********.

Bv-PrxIIF         -SEFKVSGGDHMLAQI-------------------------------------------- 199
At-PrxIIF         -SDFKVTGAEVILGQI-------------------------------------------- 201
```

Supplementary Figure 5: Alignment of the PRX subfamilies of 2-CysPRX, PRXQ, PRXIIB/E and PRXIIF in Beta vulgaris and Arabidopsis according to GenBank database. Part of the transit sequence of PRXIIE was truncated. (*) indicate full conservation among all sequences, (:) highly conservative exchanges and (.) conservative exchanges of amino acids.

Suppl. Fig. 6

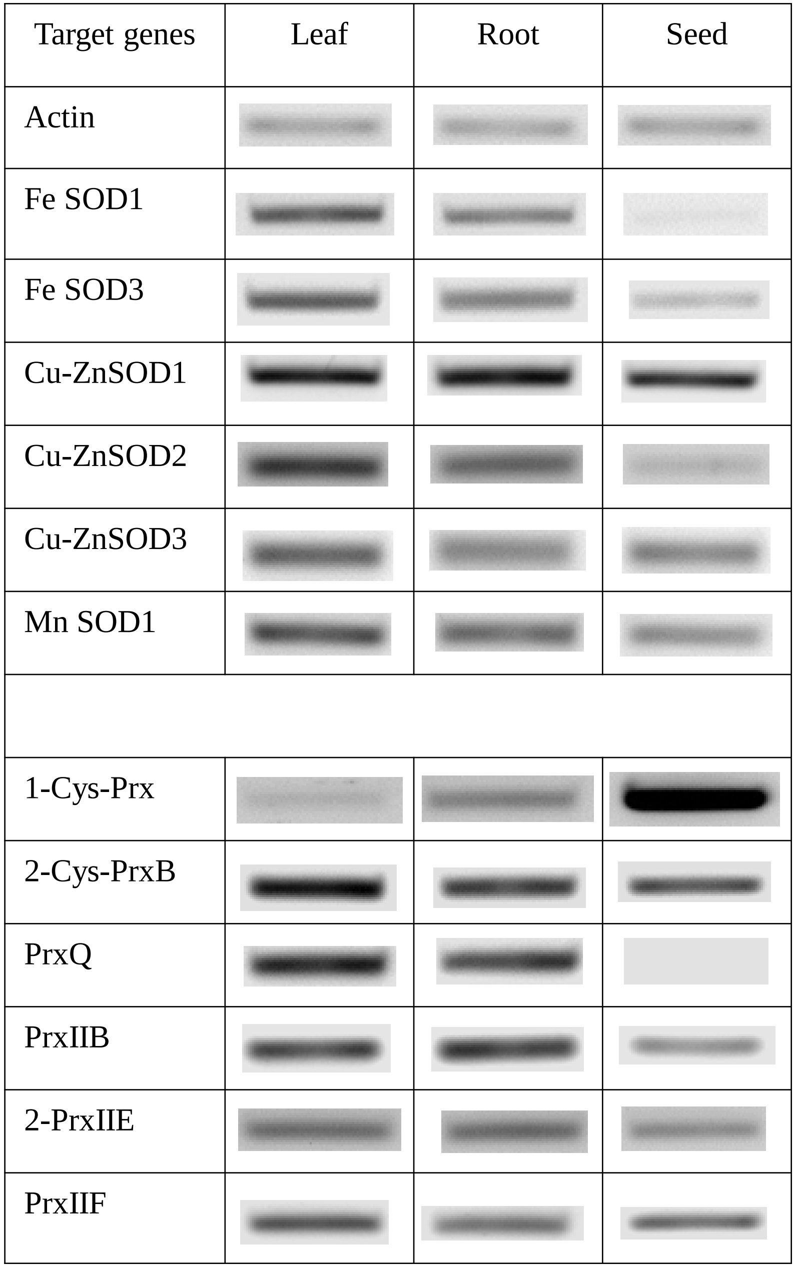

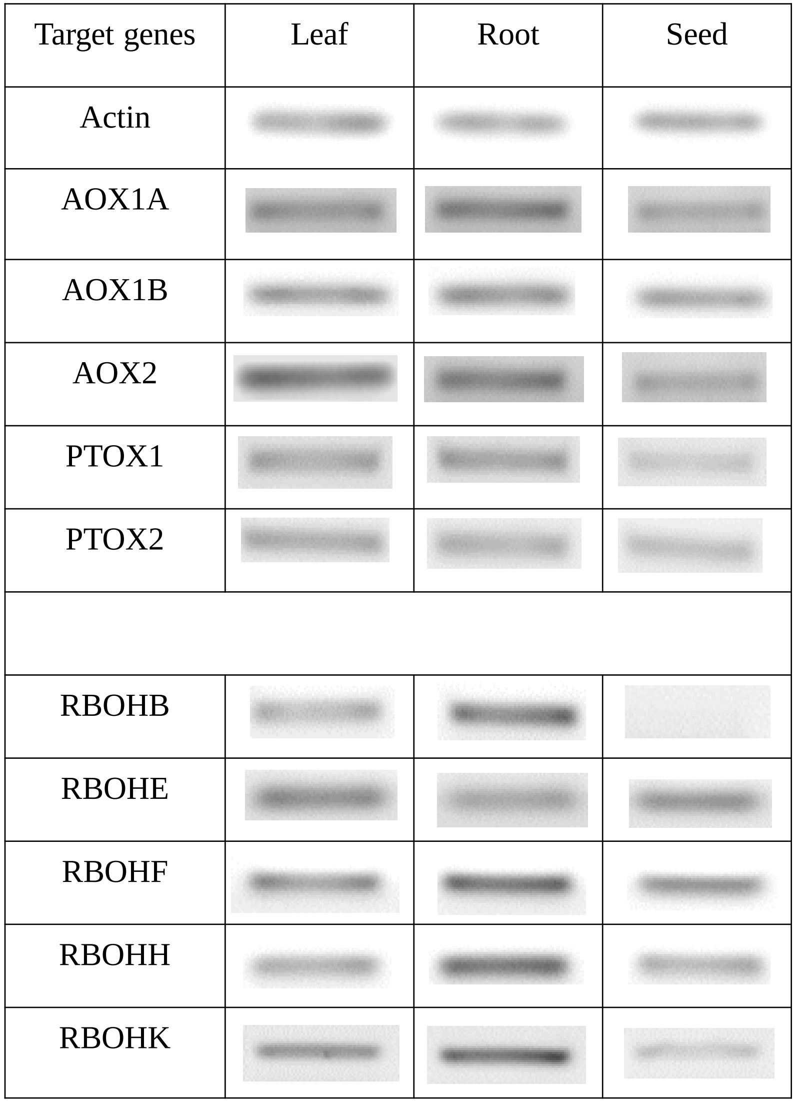

Supplementary Figure 6: Tissue-specific expression of SODs, PRXs, AOXs, PTOXs and RBOH in leaves, roots and seeds. The transcripts were amplified using gene specific primers by semi-quantitative RT-PCR from cDNA samples standardized to actin. The RT-PCR products were separated on agarose gels (1.5%) and visualized by ethidium bromide stain. The figure shows one representative result from three independent analyses.

Suppl. Fig. 7

```
BvAOX1B      ----------VSTMAITIPSGEAIHSYTEFLKEIDNGNIKNLPAPAIAIDYWR-------     54
AtAOX1D      -FLAYVISPKLAHRITGYLEEEAVNSYTEFLKDIDAGKFENSPAPAIAIDYWR-------     270
BvAOX2       -FVLYLLSPKLAHRVVGYLEEEAIYSYTEYLKDIDSGAIENVPAPAIAIDYWR-------     281
AtAOX2       -FVCYVISPRLAHRVVGYLEEEAIHSYTEFLKDIDNGKIENVAAPAIAIDYWR-------     305
BvAOX1A      -FVAYIVSPKLAHRIVGYLEEEAIHSYTEFLKEIDNGNIKNVPAPAIAIDYWR-------     305
AtAOX1A      -FLGYLISPKFAHRMVGYLEEEAIHSYTEFLKELDKGNIENVPAPAIAIDYWR-------     306
AtAOX1B      -FLGYLISPKFAHRMVGYLEEEAIHSYTEFLKELDNGNIENVPAPAIAIDYWR-------     207
AtAOX1C      -LIGYIISPKFAHRMVGYLEEEAIHSYTEFLKELDNGNIENVPAPAIAVDYWR-------     281
BvPTOX2      ISNNLGLLCCHADHFSECVENHAFETYDKFIKA-EGDELKNLPAPDVAVKYYTGGDLYLF     509
BvPTOX1      --IMYAISPRMAYHFSECVENHAFETYDKFIKA-EGDELKNLPAPDVAVKYYTGGDLYLF     277
AtPTOX       --FLYILSPRMAYHFSECVESHAYETYDKFLKA-SGEELKNMPAPDIAVKYYTGGDLYLF     266
                        :       . .*  :* :::*  .   ::*  ** :*:.*:

BvAOX1B      ------------LSSDSTLRDVVMVVRADEAHHRDVNHHFALVS------QFVPLPIS--     94
AtAOX1D      ------------LPKDATLRDVVYVIRADEAHHRDINHYASDIQFKGHELKEAPAPIG--     316
BvAOX2       ------------LPKDANLKDVITVIRADEAHHRDVNHFASDVHFQGKKLKEAPAPIG--     327
AtAOX2       ------------LPKDATLKDVVTVIRADEAHHRDVNHFASDIRNQGKELREAAAPIG--     351
BvAOX1A      ------------LSPDSTLRDVVMVVRADEAHHRDVNHFASDIHYQGRELKEAPAPVG--     351
AtAOX1A      ------------LPADATLRDVVMVVRADEAHHRDVNHFASDIHYQGRELKEAPAPIG--     352
AtAOX1B      ------------LEADATLRDVVMVVRADEAHHRDVNHYASDIHYQGRELKEAPAPIG--     253
AtAOX1C      ------------LEADATLRDVVMVVRADEAHHRDVNHYASDIHYQGHELKEAPAPIG--     327
BvPTOX2      DEFQTSRVPCSRRPIIENLYDVFVNIRDDEAEHCKTMRACQT-----HGNLRSPHSSTED     564
BvPTOX1      DEFQTSRVPCSRRPIIENLYDVFVNIRDDEAEHCKTMRACQT-----HGNLRSPHSSTED     332
AtPTOX       DEFQTSRTPNTRRPVIENLYDVFVNIRDDEAEHCKTMRACQT-----LGSLRSPHSILED     321
                              .* **.  :* ***.* .  :
```

```
AtRBOHB      DGRITGDEVKEIIALSASANKLSKIKENVDEYAALIMEELDRDNL--GYIELHNLETLLL   245
BvRBOHB      DGRITEEEVREIISLSASANKLSTILDHAEEYAALIMEELDPDGL--GFIELHNLEMLLL   288
AtRBOHD      DGRVTEEEVAEIISLSASANKLSNIQKQAKEYAALIMEELDPDNA--GFIMIENLEMLLL   327
AtRBOHA      DGRLNEAEVREIITLSASANELDNIRRQADEYAALIMEELDPYHY--GYIMIENLEILLL   295
AtRBOHC      DGRLTEDEVREIISLSASANNLSTIQKRADEYAALIMEELDPDNI--GYIMLESLETLLL   300
AtRBOHG      DGRLTEDEVREIIKLSSSANHLSCIQNKADEYAAMIMEELDPDHM--GYIMMESLKKLLL   250
AtRBOHE      DGKITREEIKELLMLSASANKLAKLKEQAEEYASLIMEELDPENF--GYIELWQLETLLL   324
BvRBOHE      DGRITRDEVQELIMLSASANRLSSLKEQAHEYANLIMEELDPENL--GYIELWQLEALLL   340
AtRBOHI      DGRITENEVKEIIILSASANNLSRLRERAEEYAALIMEELAPDGLYSQYIELKDLEILLL   330
AtRBOHF      DGRITEEEVKEIIMLSASANKLSRLKEQAEEYAALIMEELDPERL--GYIELWQLETLLL   338
BvRBOHF      DGRLTEEEVKEIITLSASANKLSRLKDQAEEYAALIMEELDPEGL--GYIELWQLETLLL   346
BvRBOHK      DGKITTDDFKQMIMLSASTNKMGMKQEEAEHFAAMIMQEIDTHQE--GYIELYQLKELFK   216
BvRBOHH      DGKLSEDEVREVLVLSASANKLNKLKTQAGEYAALIMEELDPDHQ--GYIELWQLETLLR   261
AtRBOHH      DGKLTEEEVKEVIVLSASANRLGNLKKNAAAYASLIMEELDPDHK--GYIEMWQLEILLT   269
AtRBOHJ      DGKLTEEEVKEVIVLSASANRLVNLKKNAASYASLIMEELDPNEQ--GYIEMWQLEVLLT   279
             **::.  :. ::: **:*:*.:     ..  :* :**:*:        :* : .*: *:
```

Supplementary Figure 7: Alignment of partial sequences of AOX and RBOH family in *Beta vulgaris* and *Arabidopsis* according to GenBank database. (*) indicate full conservation among all sequences, (:) highly conservative exchanges and (.) conservative exchanges of amino acids.

Suppl. Fig. 8

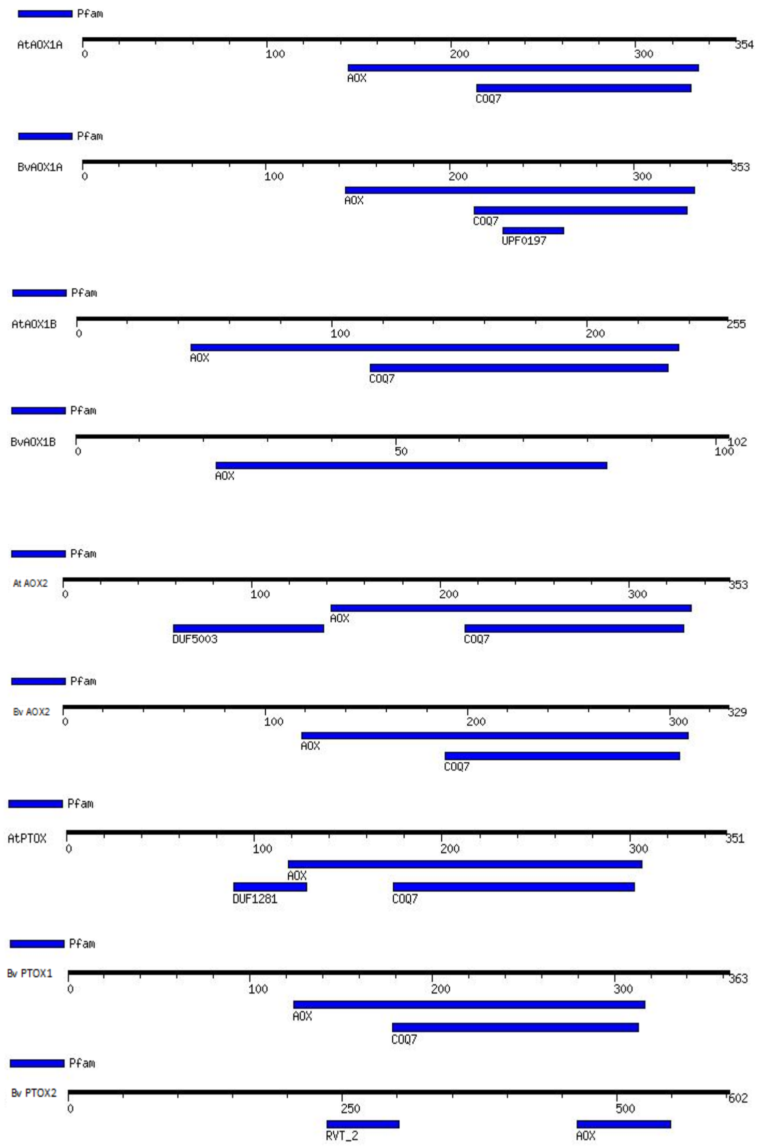

Supplementary Figure 8: Motif search of AOXs and PTOXs in *Beta vulgaris* and *Arabidopsis* according to GenBank database using http://www.genome.jp/tools/motif/.

Suppl. Fig. 9

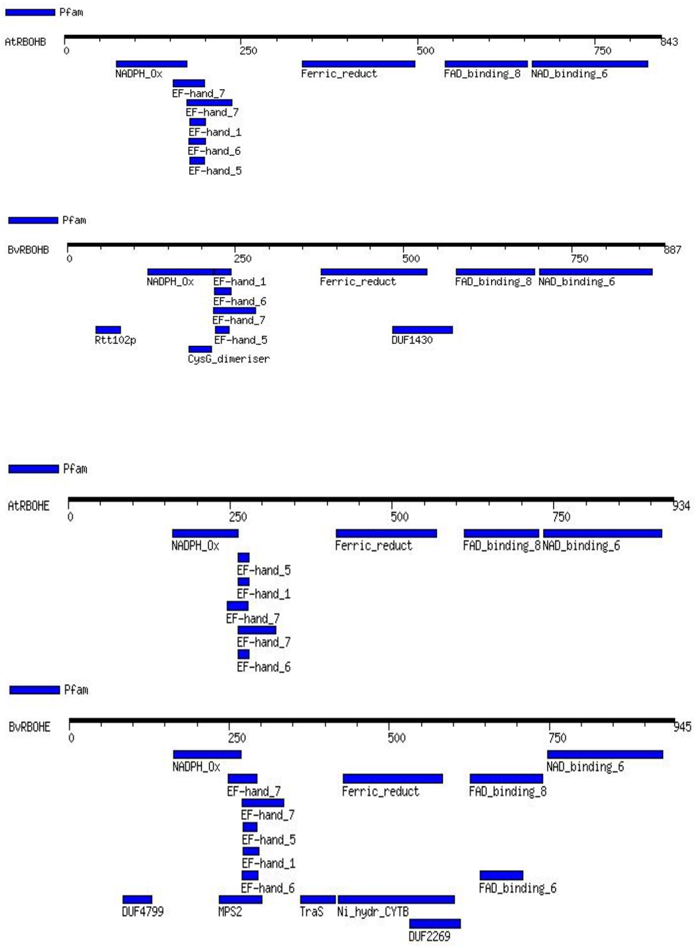

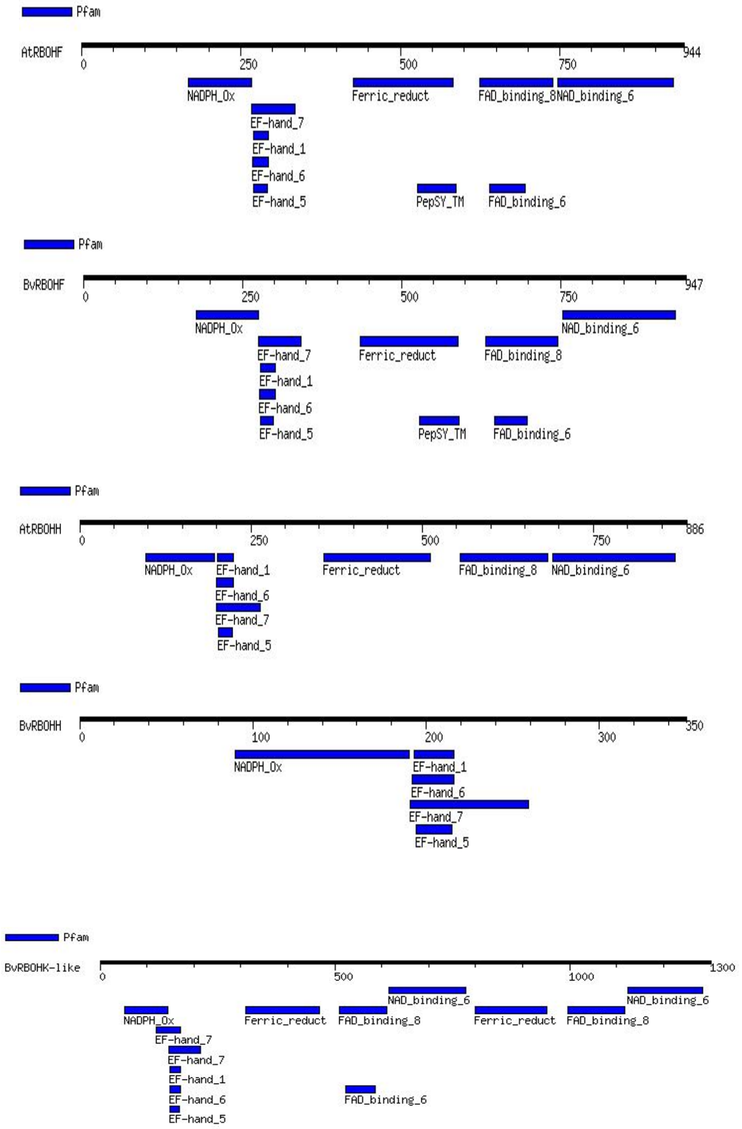

Supplementary Figure 9: Motif search of RBOH family in *Beta vulgaris* and *Arabidopsis* according to GenBank database using http://www.genome.jp/tools/motif/.
