# Supplementary material for: Redox and Reactive Oxygen Species Network in Acclimation for Salinity Tolerance in Sugar Beet
Source: J Exp Bot. 2017 Feb 18;68(5):1283–98. doi: 10.1093/jxb/erx019 (PMC5441856; doi:10.1093/jxb/erx019)
Supplement: Supplementary Data [file erx019_Supplementary_Data.zip › supplementary_Tables_S1_S5.pdf]

Table S1: Primer sequences for semi-quantitative and quantitative RT-PCR

Gene name	Reference Sequence	5´ – 3´ primer sequence
Actin	XM_010673076.1	TAAACCGAGATGGCTGATGC<br>ATACTTGGGAAGACAGCCCT
GAPDH	XM_010684569.1	GCT GCT GCT CAC TTG AAG GGT GG<br>CTT CCA CCT CTC CAG TCC TT
Fe SOD1	XM_010678412.1	TAAGAGTGTGTTCGGCACAA<br>TGGATTCCCAAAAGAACTCA
Fe SOD3	XM_010687302.1	AAAAGAAGGCCTGGAGTTGT<br>CATGGCTGCCTCTATGAACT
Cu-ZnSOD1	XM_010676650.1	TGGAAACATCTCGGGTCTTA<br>GTCTTGCTGAGCTCATGTCC
Cu-ZnSOD2	XM_010690943.1	CTTCAATGGCCTCTCTTTCA<br>AGTGACGACACCCTCAACAT
Cu-ZnSOD3	XM_010695513.1	GAAACATTGTTGCTGGCAGT<br>ACCTCGTCCAAGATCATCAG
Mn SOD1	XM_010672327.1	TTCAAAGCGCCATTAAATTC<br>CAGCTCCTTCTGCATTCATT
1-Cys-Prx	XM_010669313.1	TAGGAGATACGGTGCCAAAC<br>CAGGGTCCACCATATTCAAC
2-Cys-PrxB	XM_010685313.1	GTTTCCGATGTCACCAAGTC<br>GGGGTCTGGCTTCATAGATT
PrxIIF	XM_010696978.1	CGACAAGCTCAAGGCTAAAG<br>GAGCCTTTTCGACATTCAGA
PrxIIE	XM_010685588.1	CCCTCCTTCTTCCTCAACTC<br>GTGAGGTCGGAGATTGTGAC
PrxQ	XM_010689254.1	TTTGTTACAGTTGGACGAACCA<br>AAGTGGAAGGGGAGGAGACA
PrxIIB	XM_010693075.1	ACCTACTTGCAGTGAATGACCA<br>AGGCCTAGAGCACGAGTGTA
AOX1A	XM_010679200.1	ACGCGTACTTTGTTGCCTAC<br>AGGAACGTTCTTGATGTTGC
AOX1B	Bv_50920_agxk/<br>XP_010677502.1	TGGAGGTTGTCCTCTGACTC<br>AAGCCACATCATTTGACGAT
AOX2	XM_010692188.1	CTTTGCTGGAAGAAGCTGAG<br>GGGAAAGCAGATAAAGCACA
PTOX1	XM_010688453.1	AGGTTCTTCGTCCTGGAAAC<br>TTGGACTGATTGCATACATGA
PTOX2	Bv8_187850_zzoj/<br>XP_010686755.1	GCAGCATGTGAAGCAGTATG<br>AACTCGATATGCTTCGTTCG
RBOH B	XM_010675737.1	GCGTAGGAGAGGCAATGTTA<br>TTTGTTGGCTGATGCACTTA
RBOH E	XM_010689256.1	AGATTTTGGAGCTTGCATTG<br>TGGCCTGCTATATTCCATGT
RBOH F	XM_010678094.1	CTGCCGATCTTTACCCTGTA<br>CCCTCTGAACCAATCAAATG
RBOH H	XM_010687568.1	TTCAGGAACCCAATATCGAA<br>GCCGGATGCTTCTCATAATA
RBOH K	XM_010696961.1	TGCAGGGGAGAATGATAAGA<br>CACACCTATGCAATGACCAA

Table S2(A-D): Sequence identity within the different isoforms of the same gene group of *Beta vulgaris* subsp. vulgaris – cultivar "KWS2320".

(A) **SOD**	Bv-Fe SOD1	Bv-Fe SOD3	Bv-Cu/Zn SOD1	Bv-Cu/Zn SOD2	Bv-Cu/Zn SOD3	Bv-Mn SOD
Bv-Fe SOD1	**100**	45	57	42	0	33
Bv-Fe SOD3	45	**100**	25	22	33	35
Bv-Cu/Zn SOD1	57	25	**100**	68	73	0
Bv-Cu/Zn SOD2	46	22	68	**100**	68	36
Bv-Cu/Zn SOD3	0	33	73	68	**100**	0
Bv-Mn SOD	33	35	0	36	0	**100**

(B) **PRX**	Bv 1-Cys Prx	Bv 2-Cys Prx	Bv PrxIIB	Bv PrxIIE	Bv PrxIIF	Bv Prx Q
Bv 1-Cys Prx	**100**	32	28	26	29	24
Bv 2-Cys Prx	32	**100**	28	29	34	32
Bv PrxIIB	28	28	**100**	54	41	32
Bv PrxIIE	26	29	54	**100**	37	23
Bv PrxIIF	29	34	41	37	**100**	25
Bv Prx Q	24	33	29	23	25	**100**

(C) **AOX/ PTOX**	Bv AOX1A	Bv AOX1B	Bv AOX2	Bv PTOX1	Bv PTOX2
Bv AOX1A	**100**	97	62	26	28
Bv AOX1B	97	**100**	74	30	30
Bv AOX2	75	74	**100**	27	25
Bv PTOX1	26	30	27	**100**	99
Bv PTOX2	28	30	29	99	**100**

(D) **RBOH**	Bv RBOHB	Bv RBOHE	Bv RBOHF	Bv RBOHH	Bv RBOHK
Bv RBOHB	**100**	53	58	54	45
Bv RBOHE	53	**100**	56	51	46
Bv RBOHF	58	54	**100**	52	48
Bv RBOHH	54	52	54	**100**	44
Bv RBOHK	45	46	48	44	**100**

Table S3: Occurrence of cis-elements in the promoter regions of salinity stress-responsive genes of *B. vulgaris*. Red and bold labels indicate overrepresentation.

Upregulated

Gene name	AT-Hook	NAC/ NAM	C_2H_2 (Zn)	bZIP/ HD-ZIP	MYB	GATA	WRKY	AP2	bHLH	MADS	Trihelix	SBP	TCP	HSF	HD-PHD	DOF	ZF-HD	NF-YB	TALE	TBP	Dehydrin	BES1	Others
**Cu-ZnSOD1**	15	7	1	44	40	**40**	**25**	51	**15**	0	27	27	**0**	1	0	**21**	**11**	**35**	**6**	**9**	**5**	**1**	9
**Cu-ZnSOD2**	29	5	7	58	46	**53**	**0**	68	**21**	14	19	14	**2**	1	2	**7**	**16**	**39**	**4**	**13**	**10**	**0**	20
**Cu-ZnSOD3**	29	1	10	16	15	**38**	**1**	50	**5**	2	31	6	**6**	1	1	**15**	**0**	**33**	**5**	**15**	**12**	**0**	13
**FeSOD3**	28	2	3	27	48	**7**	**12**	71	**12**	39	32	10	**24**	0	1	**23**	**18**	**56**	**3**	**8**	**12**	**0**	10
**MnSOD1**	50	3	13	48	34	**27**	**3**	64	**31**	3	33	5	**0**	0	0	**52**	**0**	**21**	**6**	**21**	**15**	**0**	8
**2CysPRX B**	19	13	16	79	63	**92**	**4**	69	**74**	3	25	3	**8**	0	1	**36**	**15**	**55**	**4**	**8**	**13**	**4**	6
**PRX Q**	16	2	4	53	37	**15**	**1**	33	**16**	0	49	1	**0**	2	2	**41**	**19**	**36**	**4**	**9**	**25**	**0**	10
**AOX1A**	65	7	8	135	41	**37**	**35**	93	**28**	24	38	24	**4**	1	1	**44**	**24**	**33**	**7**	**12**	**19**	**2**	44
**AOX2**	45	13	6	65	49	**46**	**132**	54	**65**	0	24	32	**2**	1	1	**55**	**25**	**38**	**12**	**14**	**10**	**4**	12
**PTOX1**	29	4	3	65	48	**35**	**41**	82	**19**	6	26	9	**1**	2	0	**25**	**11**	**48**	**10**	**7**	**23**	**2**	59
**RBOH B**	24	4	5	37	16	**34**	**10**	77	**29**	2	22	8	**1**	0	1	**40**	**18**	**29**	**4**	**21**	**19**	**0**	8
**Average**	31.73	5.55	6.91	57.00	39.73	**38.55**	**24.00**	64.73	**28.64**	8.45	29.64	12.64	**4.36**	0.82	0.91	**32.64**	**14.27**	**38.45**	**5.91**	**12.45**	**14.82**	**1.18**	**18.09**
Fold enriched in Beta relative to *A. thaliana*)	0.31	0.09	0.06	1.81	0.63	**26.50**	**6.62**	0.97	**4.14**	1.24	1.37	2.67	**12.00**	0.09	0.16	∞	∞	∞	∞	∞	∞	∞	**0.12**

Down regulated

Gene name	AT-Hook	NAC/ NAM	C_2H_2 (Zn)	bZIP/ HD-ZIP	MYB	GATA	WRKY	AP2	bHLH	MADS	Trihelix	SBP	TCP	HSF	HD-PHD	DOF	ZF-HD	NF-YB	TALE	TBP	Dehydrin	BES1	Others
**1Cys-PRX**	50	2	0	56	29	**31**	**6**	74	30	6	30	5	1	4	3	**39**	**0**	**34**	**6**	**28**	**5**	**0**	19
**PRXIIB**	37	6	6	61	20	**78**	**20**	73	33	5	27	18	2	0	0	**58**	**19**	**36**	**5**	**4**	**9**	**0**	19
**PRXIIE**	31	5	9	33	34	**56**	**11**	68	14	33	28	8	2	3	3	**28**	**11**	**39**	**5**	**1**	**6**	**0**	8
**FeSOD1**	50	6	6	68	39	**48**	**10**	73	20	2	22	19	0	1	0	**32**	**40**	**40**	**10**	**18**	**4**	**0**	24
**RBOH E**	21	3	5	37	42	**41**	**3**	75	10	3	27	62	3	0	3	**48**	**19**	**35**	**5**	**1**	**5**	**0**	5
**RBOH F**	57	1	7	78	11	**36**	**10**	45	14	25	30	0	0	0	2	**24**	**22**	**31**	**2**	**19**	**7**	**0**	18
**RBOH H**	29	4	7	86	24	**50**	**38**	45	23	3	31	32	2	1	0	**44**	**17**	**45**	**8**	**3**	**14**	**0**	12
**RBOH K**	42	2	4	36	40	**102**	**24**	62	4	12	35	7	5	0	1	**55**	**20**	**30**	**10**	**19**	**8**	**0**	8
**Average**	39.63	3.63	5.50	56.88	29.88	**55.25**	**15.25**	64.38	18.50	11.13	28.75	18.88	1.88	1.13	1.50	**41.00**	**18.50**	**36.25**	**6.38**	**11.63**	**7.25**	**0.00**	14.13
Fold enriched in Beta relative to *A. thaliana*)	0.34	0.06	0.05	1.90	0.62	**44.20**	**12.20**	1.15	2.90	1.59	1.92	2.70	1.50	0.14	0.18	∞	∞	∞	∞	∞	∞	∞	0.12

Table S4: Occurrence of cis-elements at promoter region of salinity stress responsive genes of *A.thaliana*.

**Gene name**	**AT-Hook**	**NAC/ NAM**	**C_2H_2 (Zn)**	**bZIP/ HD-ZIP**	**MYB**	**GATA**	**WRKY**	**AP2**	**bHLH**	**MADS**	**Trihelix**	**SBP**	**TCP**	**HSF**	**HD-PHD**	**DOF**	**ZF-HD**	**NF-YB**	**TALE**	**TBP**	**Dehydrin**	**BES1**	**Others**
**Upregulated**																							
**Cu-ZnSOD1**	166	72	112	28	48	3		35	0	3	23	7	0	5	2	0	0	0	0	0	0	0	99
**Cu-ZnSOD2**	73	39	91	10	43	0	6	102	0	6	17	1	2	4	7	0	0	0	0	0	0	0	109
**Cu-ZnSOD3**	42	40	97	34	60	5		32	4	15	24	3	0	15	2	0	0	0	0	0	0	0	209
**FeSOD3**	57	56	130	20	63	0		49	0	10	14	3	0	11	7	0	0	0	0	0	0	0	123
**MnSOD1**	44	48	117	26	61	0	2	65	4	9	22	1	0	9	2	0	0	0	0	0	0	0	130
**2CysPRX B**	48	68	103	41	92	0	2	115	0	5	18	4	0	12	7	0	0	0	0	0	0	0	153
**PRX Q**	26	52	111	11	48	3	2	22	2	4	15	3	2	1	10	0	0	0	0	0	0	0	115
**AOX1A**	248	110	205	53	112	1	9	124	8	14	45	14	0	6	11	0	0	0	0	0	0	0	269
**AOX2**	80	53	154	41	48	3	3	57	45	4	19	5	0	8	8	0	0	0	0	0	0	0	180
**PTOX1**	157	57	105	35	62	1	4	66	2	5	20	7	0	14	1	0	0	0	0	0	0	0	126
**RBOH B**	202	81	89	47	57	0	1	64	11	0	21	4	0	10	6	0	0	0	0	0	0	0	98
**Average**	103.91	61.45	119.45	31.45	63.09	1.45	3.63	66.45	6.91	6.82	21.64	4.73	0.36	8.64	5.73	0.00	0.00	0.00	0.00	0.00	0.00	0.00	146.45
**Down regulated**																							
**Gene name**	**AT-Hook**	**NAC/ NAM**	**C_2H_2 (Zn)**	**bZIP/ HD-ZIP**	**MYB**	**GATA**	**WRKY**	**AP2**	**bHLH**	**MADS**	**Trihelix**	**SBP**	**TCP**	**HSF**	**HD-PHD**	**DOF**	**ZF-HD**	**NF-YB**	**TALE**	**TBP**	**Dehydrin**	**BES1**	**Others**
1Cys-PRX	71	84	107	27	41	4	1	48	10	8	7	4	2	9	11	0	0	0	0	0	0	0	104
PRXIIB	26	52	111	11	48	3	2	22	2	4	15	3	2	1	10	0	0	0	0	0	0	0	115
PRXIIE	221	46	115	27	63	1	0	48	6	13	16	7	0	21	2	0	0	0	0	0	0	0	136
FeSOD1	55	70	86	35	57	1	0	88	4	3	14	8	1	8	17	0	0	0	0	0	0	0	121
RBOH E	137	45	111	27	46	0	0	32	4	2	17	12	0	4	7	0	0	0	0	0	0	0	96
RBOH F	125	59	101	36	51	0	3	112	2	2	17	4	1	8	6	0	0	0	0	0	0	0	105
RBOH H	235	33	94	33	34	0	3	20	0	13	22	10	0	2	1	0	0	0	0	0	0	0	87
RBOH K	59	78	111	43	44	1	1	78	23	11	12	8	4	11	14	0	0	0	0	0	0	0	152
Average	116.13	58.38	104.50	29.88	48.00	1.25	1.25	56.00	6.38	7.00	15.00	7.00	1.25	8.00	8.50	0.00	0.00	0.00	0.00	0.00	0.00	0.00	114.50

Table S5: Sequences in details of BES1 (only present in up regulated genes of *B.vulgaris*)

**TFBS**	**Gene**	Site	Strand	Score	Sequence
**BES1**	Cu/ZnSOD1	556	+	0.87	attcCACGTtaaaa
	2CysPRX B	496	+	0.94	gggtCACGTgcgtg
		496	-	0.97	gggtcACGTGcgtg
		497	+	0.99	ggtcACGTGcg
		498	-	0.95	gtCACGTgcgt
	AOX1A	611	+	0.86	acttCACGTattct
		777	+	0.86	ggtcCACGTcagca
	AOX2	623	+	0.96	ttgaCACGTggatc
		623	-	0.99	ttgacACGTGgatc
		624	+	0.97	tgacACGTGga
		625	-	0.97	gaCACGTggat
	PTOX1	441	-	0.86	tcaatACGTGgcca
		666	-	0.88	ttgagACGTGttgt
